# Tumor location and neurocognitive function—Unravelling the association and identifying relevant anatomical substrates in intra-axial brain tumors

**DOI:** 10.1093/noajnl/vdae020

**Published:** 2024-02-09

**Authors:** Kanchi Shah, Vinayak Bhartia, Chandrima Biswas, Arpita Sahu, Prakash M Shetty, Vikas Singh, Parthiban Velayutham, Suyash P Awate, Aliasgar V Moiyadi

**Affiliations:** Neurosurgical Services, Department of Surgical Oncology, Tata Memorial Center, Mumbai, Maharashtra, India; Department of Health Sciences, Homi Bhabha National Institute, Mumbai, Maharashtra, India; Computer Science and Engineering Department, Indian Institute of Technology (IIT) Bombay. Mumbai, Maharashtra, India; Neurosurgical Services, Department of Surgical Oncology, Tata Memorial Center, Mumbai, Maharashtra, India; Department of Health Sciences, Homi Bhabha National Institute, Mumbai, Maharashtra, India; Department of Health Sciences, Homi Bhabha National Institute, Mumbai, Maharashtra, India; Department of Radiodiagnosis, Tata Memorial Center, Mumbai, Maharashtra, India; Neurosurgical Services, Department of Surgical Oncology, Tata Memorial Center, Mumbai, Maharashtra, India; Department of Health Sciences, Homi Bhabha National Institute, Mumbai, Maharashtra, India; Neurosurgical Services, Department of Surgical Oncology, Tata Memorial Center, Mumbai, Maharashtra, India; Department of Health Sciences, Homi Bhabha National Institute, Mumbai, Maharashtra, India; Neurosurgical Services, Department of Surgical Oncology, Tata Memorial Center, Mumbai, Maharashtra, India; Department of Health Sciences, Homi Bhabha National Institute, Mumbai, Maharashtra, India; Computer Science and Engineering Department, Indian Institute of Technology (IIT) Bombay. Mumbai, Maharashtra, India; Neurosurgical Services, Department of Surgical Oncology, Tata Memorial Center, Mumbai, Maharashtra, India; Department of Health Sciences, Homi Bhabha National Institute, Mumbai, Maharashtra, India

**Keywords:** gliomas, neurocognition, tumor localization maps, voxel-based mapping

## Abstract

**Background:**

Neurocognitive function is a key outcome indicator of therapy in brain tumors. Understanding the underlying anatomical substrates involved in domain function and the pathophysiological basis of dysfunction can help ameliorate the effects of therapy and tailor directed rehabilitative strategies.

**Methods:**

Hundred adult diffuse gliomas were co-registered onto a common demographic-specific brain template to create tumor localization maps. Voxel-based lesion symptom (VLSM) technique was used to assign an association between individual voxels and neuropsychological dysfunction in various domains (attention and executive function (A & EF), language, memory, visuospatial/constructive abilities, and visuomotor speed). The probability maps thus generated were further co-registered to cortical and subcortical atlases. A permutation-based statistical testing method was used to evaluate the statistically and clinically significant anatomical parcels associated with domain dysfunction and to create heat maps.

**Results:**

Neurocognition was affected in a high proportion of subjects (93%), with A & EF and memory being the most affected domains. Left-sided networks were implicated in patients with A & EF, memory, and language deficits with the perisylvian white matter tracts being the most common across domains. Visuospatial dysfunction was associated with lesions involving the right perisylvian cortical regions, whereas deficits in visuomotor speed were associated with lesions involving primary visual and motor output pathways.

**Conclusions:**

Significant baseline neurocognitive deficits are prevalent in gliomas. These are multidomain and the perisylvian network especially on the left side seems to be very important, being implicated in dysfunction of many domains.

**Key Points**

- Tumor localization maps were generated in Indian subjects using demographic-specific templates.
- Voxel symptom lesion mapping correlated tumor location and neurocognitive domain dysfunction.
- Affection of the left perisylvian network emerged as the cause of multiple domain dysfunction.

**Importance of the Study**

MRIs of 100 diffuse gliomas were mapped onto a common MRI template (Indian Brain Template) to generate tumor localization maps depicting the distribution of tumor location. The probabilities of a tumor being located at a specified voxel were compared between subjects with and without affection of specific neurocognitive domains (attention/executive function (A & EF), language, memory, visuospatial/constructive abilities, and visuomotor speed) using a rigorous permutation-based statistical test. Significant regions above a threshold (*P* < .05 was considered statistically significant, but *P* < .2 was considered clinically significant) were selected and using cortical and subcortical atlases co-registered to the test data, named parcels were identified. Besides known cortical substrates, overlapping regions of the left-sided perisylvian white matter tracts were found to be associated with A & EF, memory, and language dysfunction.

Neurocognitive function (NCF) is a key performance indicator in brain tumor patients. Treatment strategies (such as awake surgery, hippocampal sparing radiotherapy) and rehabilitation programs are customized based on NCF and it also serves as an important outcome measure to assess the impact of these interventions. Individual domains of NCF represents a set of functions which is believed to be catered to by specific regions of the brain. Traditionally, these functions have been assigned broadly to the major lobes of the brain^1^ and this association is well reported.^2,3^ As our understanding of brain connections evolves, it is increasingly clear that individual domain function is related to specific anatomico-functional units linked by unique interconnected fiber networks across lobes.^4–9^ Voxel-based lesion symptom mapping has been used in neuroscience research to correlate anatomical regions with disease states which result in loss of function, and thereby indirectly to infer functions of these anatomical substrates.^10,11^ Besides providing invaluable information regarding anatomico-functional correlation at the individual level, the technique can be utilized to create population-based maps and atlases and is a powerful method to discern patterns and identify networks which are common across groups. Tumor localization maps (TLM) help understand patterns of distribution of tumors within populations and provide insights into how this affects neurological and neurocognitive function.^12–15^ Maps developed using the technique of voxel symptom lesion mapping (VSLM) enable ascribing independent association of the effect being studied (eg, neurocognitive dysfunction) to an individual voxel and avoid grouping regions based on anatomical lobes alone.

Neurocognitive dysfunction in brain tumors at baseline is well described.^2,16^ However, TLMs correlated with NCF have not been reported widely.^17^ Specifically for the Indian population, no such data exists. As NCF normative data varies across populations, understanding NCF using TLMs specific to the population is critical. Anatomical brain templates serve as a reference for generating such maps and atlases, allowing data to be brought into a common space for group-level comparisons and correlations. Internal and external validity of these maps is best when the study population and the template population are similar. The Montreal Neurological Institute (MNI) brain is the most common brain template used in neuroimaging studies.^18–21^ The anthropometric features of the brains of Indian subjects differ from the Caucasian brain on which MNI is based. Recently, the Indian Brain template has been developed and it is most suitable for generating population specific maps for Indian subjects.^22^ In the present study, we report the first ever tumor localization maps generated for Indian subjects with gliomas and correlate them with domain level neurocognitive function to identify critical cortical and subcortical substrates related to cognitive dysfunction.

## Methodology

This study was conducted in the neurosurgical oncology department of a tertiary care oncology hospital. It was approved by the Institutional Ethics Committee (IEC no. 3882) with a waiver of consent (as the study was retrospective and used previously acquired data collected as part of routine care). All consecutive adult patients with histologically proven adult-type diffuse gliomas (as per WHO 2021 classification) that underwent craniotomy and excision between January 2019 and October 2021 were screened. As a routine practice in the department, detailed neuropsychological assessment (NPA) and a brain tumor protocol MRI are performed for all tumors planned for elective surgery. The relevant clinical, radiological, and histopathological information was obtained from the neurosurgical database, the hospital’s electronic medical records (EMR), and the PACS system. Histology was recorded from the routine reports, including IDH molecular status (immunohistochemistry and sequencing to confirm the presence or absence of IDH mutations as part of routine practice) as per the WHO 2021 classification.

### Neuropsychological Evaluation

The Addenbrooke’s Cognitive Examination (ACE-III) in Indian English and Hindi was used as the primary screening tool for all patients,^23^ and data on handedness and educational status were also recorded.^24^ Following the screening, an extensive neuropsychological test battery (minimum 2 tests per domain) was administered to evaluate the patients’ neurocognitive functioning (NCF) in 5 major cognitive domains, including attention and executive functions (A & EF), memory, language, visuospatial/visuoconstructive function, and visuo-motor speed as per our standard protocol published earlier^2^ (Supplementary Material 1). Standardized tests were used wherever possible, but some tests were modified to suit the diverse age, literacy, cultural, and socio-economic backgrounds of the patients.^25^ The performance for each test was recorded as normal, mild-moderate, or severe, based on *z*-scores [severe (*z* < −2 SD), mild to moderate (−2 SD > *z* > 0), and normal (*z* > 0)] or subjectively graded^26^ for tests where standardized *z-*scores could not be computed. A domain was considered affected if any one of the tests pertaining to that domain was abnormal, with severity categorized based on the worst test result. The results of ACE were not considered for interpreting domain dysfunction, although it covers many of the domains. For this study, only severe deficits were considered to classify a domain as “affected.” All others were categorized as a “control” group for the domain.

### MRI Evaluation

Preoperatively, a detailed MR evaluation (“brain tumor protocol”—Supplementary Material 3) was routinely performed at the center within the 2 weeks prior to the scheduled surgery. This protocol included T1, T2, FLAIR, and post contrast T1 sequences. Volumetric sequences were used wherever available. Anonymized DICOM images were converted into the NIfTI using the dcm2nii (µm) application and saved as a nii.gz file for the purpose of segmentation. The tumor was segmented on T2 MRI sequences using the polygon selection and interpolation function in the ITK-SNAP application, which can be accessed at this link^27^ (www.itksnap.org). The images were carefully reviewed by 1 of the authors (KJ) and segmentation was done on both T1 and T2 images to include all the T2 abnormality. FLAIR images were used for visually correlating with the T2 images for each patient. This also provided a volumetric assessment of tumor mass which was used for the analysis. Once segmented, 2 senior investigators independently reviewed the segmentations (AM, a senior neurosurgeon with more than 15 years experience in neuro-oncology; and AS, senior neuroradiologist with 10 years experience) and made any necessary adjustments to the segmented volumes. Discordance (noted in 18 cases) was resolved by mutual discussion and consensus. After this verification, the segmented volumes were exported to a drive folder and saved in NIfTI image format for further analysis.

### Image-Registration Procedure

To create the maps, all MRI scans were registered in a standard anatomical space. For this purpose, we employed the Indian Brain Template (IBT),^22^ which has been demonstrated to provide superior alignment and accuracy in the analysis of brain images of Indian patients compared to other standard brain atlases such as the Montreal Neurological Institute (MNI) template.^28–30^ The C4 age group of the IBT (26–40 years group), was used as it matched the age range of our patients.

We used T2-weighted MRI sequences for tumor segmentation as they provide superior contrast and sensitivity to edema, infiltration, and other biological factors associated with tumorigenesis compared to T1 sequences.^31^ However, T1-weighted images were more suitable for image registration as they offer greater anatomical detail and, more importantly, provide significantly higher similarity to the intensities of the IBT image which is also a T1-weighted MR image.^31^

**Figure 1.**
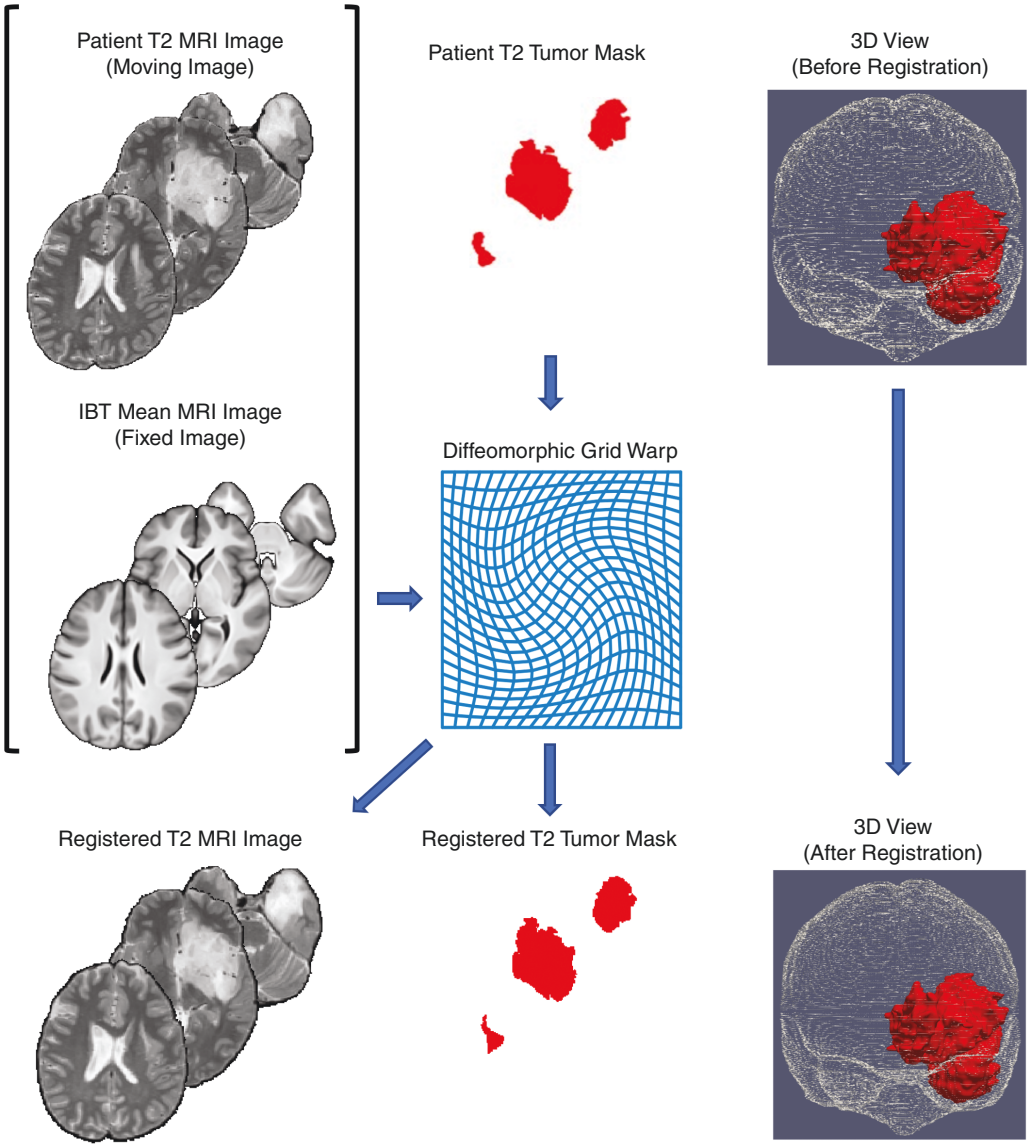
Diffeomorphic registration for brain and tumor localization. Diffeomorphic registration aligns the patient’s brain to the Indian Brain Template (IBT), establishing a precise mapping of anatomical structures to the standardized IBT space. T2-weighted MRI is registered to IBT space via a 2-step process. First, it is registered to its corresponding T1-weighted MRI space. Then, the diffeomorphic grid warp from registering T1-weighted MRI to IBT space is utilized to register T2-weighted MRI to IBT space. The same diffeomorphic grid warp is employed to register the associated tumor within the IBT space, facilitating accurate localization and visualization of the tumor in the standardized brain template.

The outline of the methodology for image registration is depicted diagrammatically in Figure 1. Before image registration, all the images were resampled to a voxel size of 1 mm × 1 mm × 1 mm and the Brain Extraction Tool (BET)^32^ was used for automated brain image extraction from individual native MR images. First, each patient’s T1 MRI image with tumor was registered to the IBT MRI image (which does not contain any tumor). To achieve accurate registration, the tumor segmentation for the patient image present in the T1 MRI was used as a registration mask and this was excluded from the patient MRI. Next, we used dense diffeomorphic registration, a type of nonlinear/deformable image registration that produces spatially smooth and anatomically plausible deformations. The diffeomorphic warp obtained after the registration of each MRI image was saved for future requirements. Specifically, G_i_ denoted the warp from the T1 MRI of the *i*th patient to the IBT image. During image registration, we employed the normalized-cross-correlation between patches as the similarity metric.^33^ Then, we used rigid registration to align the T2 MRI image of every patient with the corresponding T1 MRI image for the same patient. We used this rigid registration to transform the tumor segmentation from the T2 MRI’s spatial coordinate frame to the T1 MRI’s spatial coordinate frame. Finally, for each patient, we applied the grid warp G_i_ to transform the tumor segmentation from each patient’s T1 MRI spatial coordinate frame to the IBT image’s spatial coordinate frame. After the tumor segmentation (binary) for each patient was transformed to the IBT’s spatial coordinate system, the resulting tumor segmentation takes fuzzy values in the range [0, 1]; such a fuzzy value at any chosen voxel can be regarded as the probability of that chosen brain voxel lying within the tumor, for that patient, within the IBT coordinate system. We repeated this process for all 105 patients to obtain all 105 tumor segmentations registered to the IBT anatomical space. This yielded tumor localization maps for both T1 and T2 images.

### Hypothesis Testing and Statistical Analysis

**Figure 2.**
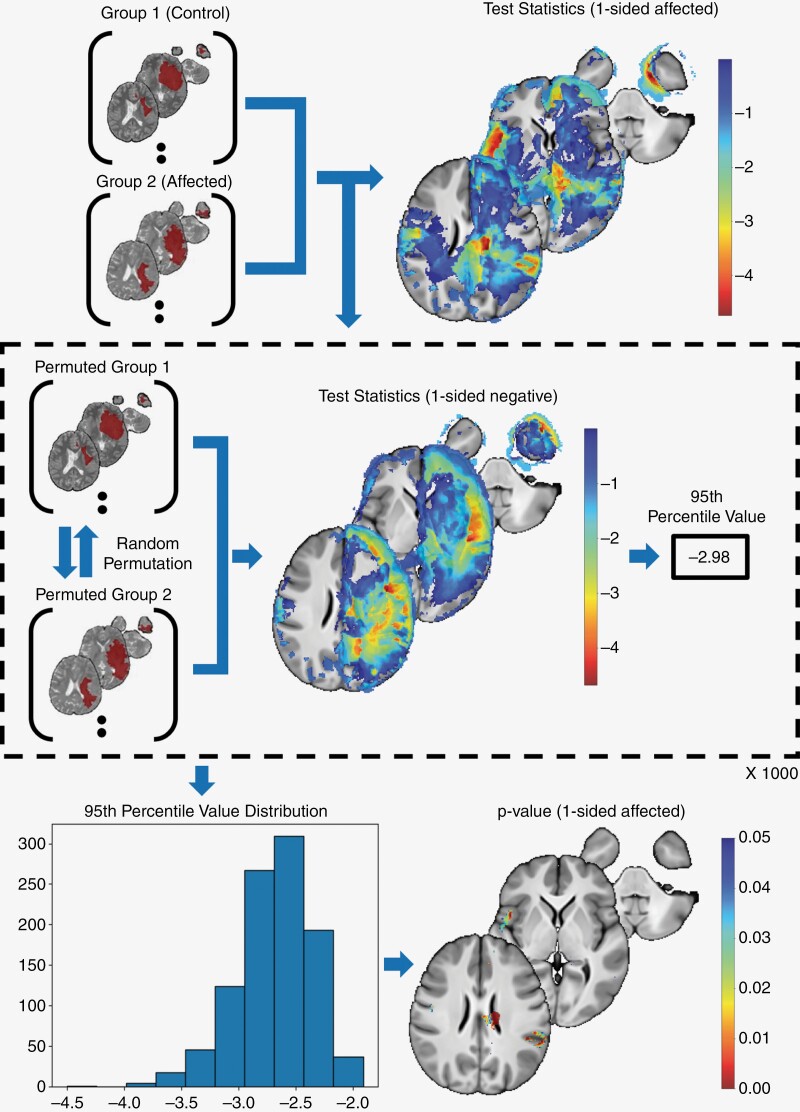
(Perm95) 95th percentile permutation test to compare group means. The test follows a 1-sided permutation approach, with the null hypothesis that the mean of the control group is greater than the mean of the affected group. Random permutations are performed, exchanging group labels, and the *t*-statistic is calculated for each permutation. The 95th percentile value is extracted from the resulting values of test statistics, forming the null distribution. This process is repeated for 1000 permutations, generating a null distribution of 95th percentile values. The original *t*-statistic value obtained prior to permutations is compared to the null distribution, yielding a *P*-value based on the number of permutations smaller than the observed test statistic.

To test the hypothesis, the subjects were divided into 2 groups based on any chosen NCF domain. The 2 groups were (i) the control group that included all patients with less than severe deficits (including mild/ moderate or no impairment) and (ii) the affected group that included patients with severe impairment in the particular domain. For each group, at each voxel in the brain, we assumed a distribution of tumor probabilities across patients in the group. Then, at each voxel, we considered the null hypothesis that the distribution of tumor probabilities in the control group was identical to the distribution of tumor probabilities in the affected group. Subsequently, we performed statistical tests of the hypothesis at every voxel in the brain, initially using a parametric test, ie, a *t*-test. We aimed to study the relationship between the presence of the tumor in those brain locations in the affected group where the tumor was absent in the control group for each domain of NCF. Therefore, a 1-sided hypothesis test for negative values of the *t* statistic was analyzed with an alpha level of 0.05. While the *t*-test is a popular statistical method used to compare the means of 2 groups, it has 2 critical limitations relevant to our study. Since the *t*-test is a parametric test and fails to account for false positives resulting from multiple comparisons, it has limitations. Therefore, we adopted a non-parametric test using a permutation-testing approach. Compared to the *t*-test, permutation testing^34^ provides a more general non-parametric approach that does not require strong assumptions about the underlying distribution of the data. Instead, the permutation test estimates the null distribution of the test statistic using a data-driven approach that first assumes exchangeability under the null hypothesis, then leverages exchangeability to randomly re-assign/permute the group labels, computes the test-statistic value under each random assignment, and finally produces a histogram of test statistics. By comparing the value of the observed test statistic to the empirical distribution, we obtain a *P*-value. When the null hypothesis is true, the empirical distribution of the test statistic estimates the theoretical distribution well, the observed value of the test statistic will be similar to those in the empirical distribution, and hence the resulting *P*-value will tend to be large. This ensures a stringent control on false positives but can exclude potentially relevant effect in smaller samples. Permutation testing also allows us to control for false positives arising from multiple comparisons across multiple brain voxels. Under the null hypothesis, we built a histogram of the test statistic using, in each permutation, the minimum of the set of test-statistic values across all brain voxels. Such a test-statistic histogram empirically accounts for extreme values of test statistics occurring by chance across the brain. The resulting histogram was used to compute the *P*-values at each voxel based on the observed test-statistic values. Building the null distribution of the test statistic using the minimum test statistic across brain voxels provides strong control over false positives arising from multiple comparisons. Due to this stringent control, very large sample sizes are needed to be able to produce sufficiently small *P*-values indicating statistically significant differences between the groups. To handle this limitation, for building the null distribution of the test statistic, we replaced the use of the minimum test statistic (across all brain voxels) with the use of the 5th percentile of the set of test statistics across all brain voxels (permutation 95th percentile or Perm95). This provided a reasonable tradeoff between the strong control over the false positive rate to gain the ability to detect statistically significant differences even with the sample sizes in our study. We used 1000 permutations to build the null histogram and a *P* value cutoff of 0.05 to identify statistically significant voxels for creating the maps. However, considering the exploratory nature of the study and the relatively small sample, we also evaluated maps generated with *P*-values cutoff of 0.2 to create the heat maps of the cortical and subcortical parcels. Figure 2 shows the Perm95 testing methodology performed.

In addition, we also conducted a per-voxel permutation test using the relative risk as the test statistic to analyze the presence of tumors, as has been described before by Habet’s et al.^17^ The relative risk was calculated as the ratio between the number of patients having tumor in the affected group to those in the control group. To determine if a patient had a tumor in a particular voxel, we applied a threshold value to the tumor mask (after registration), assuming it to be 0.03 in our case. Subsequently, per-voxel randomization was performed and *q-*values (false discovery rate) were calculated as previously described.^17^

### Creation of Cortical and Subcortical Atlases

To interpret the locations of the voxels that produced small *P*-values, we mapped these voxels to parcels in standard anatomical atlases. We used 2 atlases, 1 each for cortical and subcortical parcels. We used the cortical parcellation provided with the Indian Brain Template (IBT) that includes a standardized and validated atlas of cortical regions based on the anatomical variability observed in the Indian population.^22^ This parcellation consists of 200 cortical regions defined by gyral and sulcal anatomy. In addition, we used a subcortical region parcellation, provided by the neuroanatomy toolbox for Brainstorm (https://neuroimage.usc.edu/brainstorm), referred to as the tractography-based atlas of the human brain. This parcellation includes different networks including projection, association, and commissural fibers. We used 2 thresholds for *P* value (.05 and .2) from the Perm95 test to designate a parcel as involved. Depending on the threshold, the involvement of the parcel was quantified in terms of percentage involvement of the parcel by tumor in the affected group as compared to the control group and the top 15 were reported. In reporting clinically relevant parcels, we have also considered that parcels of anatomical contiguous regions may be co-contributory in a particular domain (dys)function, even though percentage involvement at statistically significant thresholds may be variable. The entire cohort of diffuse gliomas was included in the analysis and is reported in the manuscript. As a supplementary analysis, we also analyzed the TLMs and probability maps for GBMs and LGGs separately, details of which are available in Supplementary Materials 13 and 14.

## Results

### Clinicodemographic Profile

A total of 100 cases were included. The median age of the population was 40.5 years. Supplementary Table 2 depicts the overall profile of the group. There were slightly more diffuse lower grade IDH-mutant gliomas (57) than glioblastomas (43). Left-sided tumors predominated (69%). Most subjects had at a least basic school level of education. Seventy seven percent had no prior history of any treatment in the index region. Frontal and insular region were the commonest lobes involved and 44% were multi-lobar with the fronto-temporo-insular region showing the highest involvement. The average volume was 90.8 cc.

### Neurocognitive Function

The majority of the subjects (93%) had overall NCF affected at baseline. The domain-wise affection was however variable. Attention and EF (78%) as well as memory (63%) were the most frequently affected domains, closely followed by visuomotor speed (51%) and visuospatial ability (46%). Language was the least affected domain (25%).

### Correlation of Tumor Location and NCF

**Figure 3.**
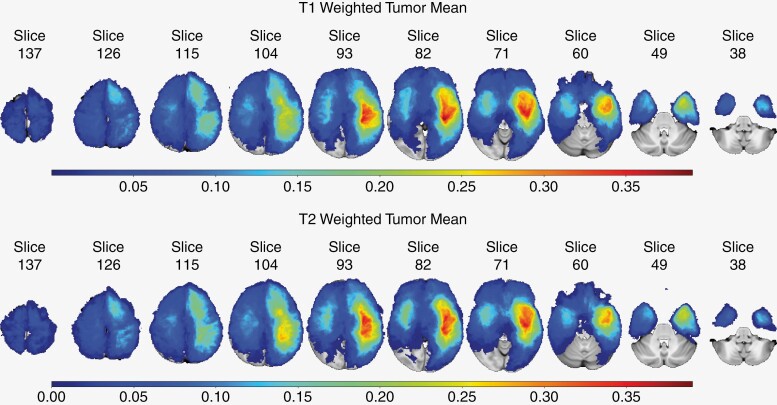
Tumor mean distribution in T1 and T2 MRI scans. The figure displays the mean tumor distribution across the entire cohort of patients. Higher values indicate a significant proportion of patients with tumor occurrence in that specific region of the brain.

**Figure 4.**
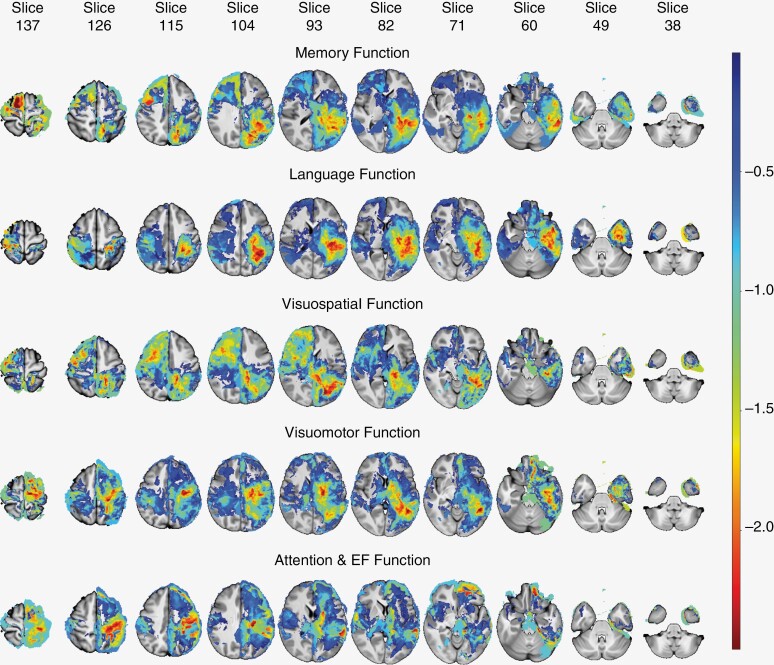
Negative *t*-statistic values for all neurocognitive functionality (NCF). The figure displays negative *t*-statistic values for each NCF measure, highlighting regions where the tumor mean of the control group is smaller than that of the affected group. These findings suggest significant 1-sided differences in tumor presence between the2 groups based on particular NCF.

**Figure 5.**
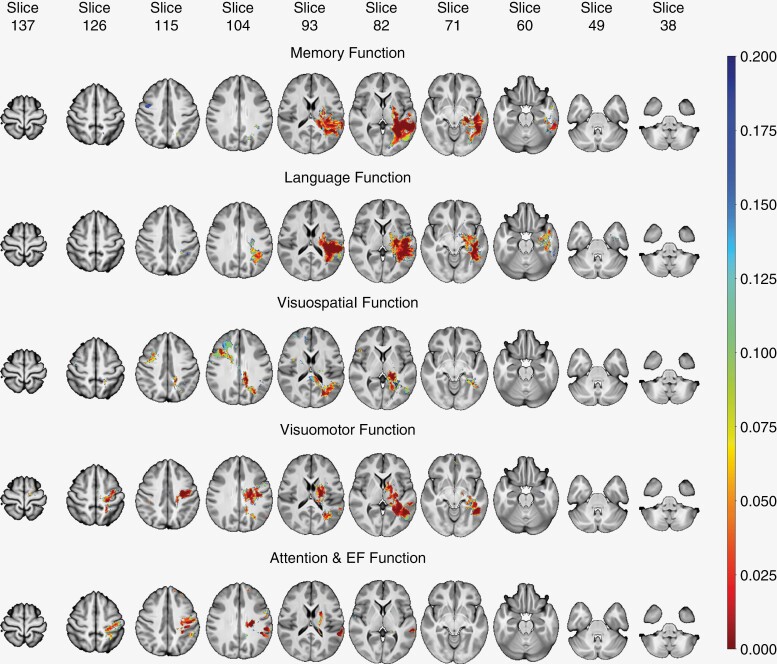
Perm95-test results for all neurocognitive functionality (NCF). The figure showcases brain slices representing different NCF measures, with accompanying *P*-values obtained from the Perm95-test analysis. The output is thresholded at alpha (*P*) = 0.2, and only values below this threshold are displayed, highlighting brain regions with statistically significant differences in NCF between the control and affected groups. Note that *P*-values <.05 are represented by orange-red colored regions.

Figure 3 depicts the tumor localization map (TLMs) of the study group, for T1 and T2 images. Figure 4 shows the effect size (using the 1-sided *t*-test statistic) and Figure 5 depicts the Perm95 (*P* value < .2) map which also includes voxels with *P* < .05 (color range orange to red). A separate map showing parcels with only *P* < .05 significance is provided in Supplementary Material 4. Parametric 1-sided *t*-test (*P* < .05) and *q-*value maps (0.2 and 0.4 thresholds), based on relative risk assessment are provided in Supplementary Materials 5–8. The Perm95 maps were found to be the most stringent and we used these for generating the voxel-based heat maps.

#### Heatmaps of putative cortical and subcortical parcels involved in individual domain dysfunction.—

**Figure 6.**
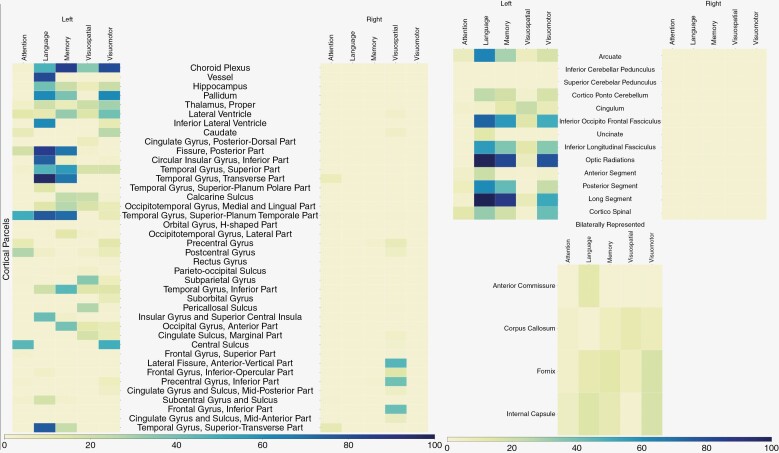
Heatmaps of significant cortical parcels (left side of figure) and subcortical parcels (right half of figure) implicated in various neurocognitive domain (*x*-axis) dysfunction. All subcortical parcels and top 40 cortical parcels which are predominantly covered by statistically significant voxels are shown. Each cell within the heatmap indicates the percentage of a specific cortical region that is covered by the significant voxels, obtained through a Perm95-test analysis with a *P*-value threshold of 0.2. The heatmaps provide a visual representation of the distribution and magnitude of statistically significant differences in NCF across the most prominently affected parcels.

Figure 6 depicts the heat maps created using a *P* value of 0.2 (heat maps with *P* < .05 threshold and a comparative list of 15 most affected parcels is provided in Supplementary Materials 9 and 10). The heat maps for *t*-test and *q-*value maps were also generated (data not shown) and showed significant overlap with the Perm95 maps which were then used for the final analysis. Based on the heat maps the following observations were made.

#### Attention and executive function (A & EF).—

Left-sided regions were more involved in A & EF. Left superior temporal, peri-rolandic frontal, and inferior parietal areas were most often involved. Additionally, the right sided superior temporal gyrus and peri-rolandic region involvement was also seen. Subcortically, corresponding to cortical parcels, the left-sided fibers predominantly the corticospinal projection fibers, and perislyvian networks were found to be involved.

#### Language.—

Almost exclusively, left-sided parcels were involved in subjects with language dysfunction. The perislyvian and opercular cortical regions (inferior frontal, superior temporal, and inferior parietal lobule) as well as parts of the insula and medial temporal lobe including hippocampus were predominantly involved. Subcortically, as expected, perislyvian networks including the arcuate complex and inferior fronto-occipital fasciculus (IFOF), the inferior longitudinal fasciculus (ILF) as well as optic radiations and the CST projection system were involved.

#### Memory.—

This showed significant overlap with language parcels. Predominantly, left occipitotemporal regions were seen to be involved in memory dysfunction. This included many temporal neocortical regions besides the hippocampus. In addition, thalamus and basal ganglia also were identified. The parcel labeled “choroid plexus” could be an artifact of the image registration procedure and probably indicates deep periventricular tracts including fornix and the Papez circuit. The fornix was also seen to be significant on the subcortical parcellation, besides cingulum and the posterior perislyvian segment of the arcuate, optic radiations and the ILF.

#### Visuospatial function.—

Right sided cortical regions including anterior perislyvian regions (including the inferior frontal gyrus—IFG), cingulate, and peri-callosal areas were predominantly implicated. In addition, the temporal lobe regions and in particular the left occipitotemporal gyrus was also identified. In contrast, at the subcortical level, more left-sided fibers (ILF, IFOF) were identified though cingulum, arcuate, and CST were identified bilaterally.

#### Visuomotor speed.—

Left occipitotemporal region (visual recognition area) and peri-Rolandic (especially precentral gyrus) cortex was predominantly involved and subcortically, the left arcuate, ILF, cingulum as well as corpus callosum (indicating bilateral processing) and CST projection fibers were implicated.

## Discussion

Our study employed tumor localization maps (TLMs) created from a large cohort of intrinsic brain tumors, specifically in Indian subjects and identified preferential regions of involvement of gliomas. Further, by co-registering the TLMs to cortical and subcortical atlases we have been able to identify anatomical substrates closely associated with various cognitive domains, corroborating much of our existing knowledge, and shedding new insights onto lesser-known associations.

Very few contemporary studies have examined the association of tumor location with NCF using TLMs. Habet’s et al.^17^ described a large series of 72 cases of gliomas in the Caucasian population. We did not find any such similar study in other populations. Whereas the broad methodology we have employed is similar, our work differs from their study in several key aspects. We had a preponderance of higher-grade tumors which were larger in size. One of the reasons could be that we considered all T2-FLAIR abnormalities in calculating the volume of the lesion for both LGG and glioblastomas. Though the T2/FLAIR signal abnormality is widely accepted as the gold standard for delineating LGG, many previous studies have used only post contrast T1 volume for glioblastomas. However, mounting evidence now shows that even in glioblastomas, tumor extends far beyond the contrast enhancing zone.^35^ In fact, the T2 FLAIR region is the target for surgical resections and its removal associated with a better prognosis. Therefore, the T2 FLAIR region more accurately represents the tumor and that is also the reason we have used it to delineate both, LGG and glioblastomas. Probably because of larger tumors in our series, and a difference in the tests used and their interpretations, our cohort had a higher proportion of severely affected domains (almost double of what Habets et al. reported). We have earlier reported a very high prevalence of baseline neurocognitive deficits in our population of glioma patients.^2^ For the present analysis, we considered only severe affection as abnormal. NCF is a continuum of function based on normative comparators and the distinction between normal and mild affection can be very blurred, whereas severe deficits can be more reliably defined. Further, there is a wide variability in literature as regards the cutoffs and definition for cognitive dysfunction.^36^ By using only severe cognitive dysfunction as the cutoff in our analysis, we have ensured a better discrimination between the groups, though we accept that the sensitivity could have reduced. The larger proportion of severely affected subjects also gave us a higher rate of true positivity in the statistical analysis. We also used the cortical parcellation atlas provided with the IBT which has a larger number of annotated parcels. Finally, our statistical methodology for the hypothesis testing differed from that described by Habets et al. per-voxel testing is susceptible to a large number of false positives (FPs) (voxels may be incorrectly identified as significant). To address this issue, *q*-values are used to control the false discovery rate (FDR) as was reported by Habets et al.^17^ The *q*-value is a measure that adjusts the original *P*-values obtained from the per-voxel permutation test, resulting in a more stringent set of values. Though *q*-values can reduce the likelihood of FPs, there are certain limitations associated with it. Firstly, the computation of relative risk relies on thresholding the per-voxel tumor probabilities, which in turn introduces some degree of uncertainty and can undermine the reliability of the results. In comparison, the *t*-statistic offers a more dependable approach for statistical analysis as it does not require such thresholding. Secondly, *q*-values are computed based on the distribution of *P*-values and hence exhibit a considerable dependency on the specific region under investigation and the results may vary significantly depending on the region being analyzed, which hampers their reliability as a robust statistical measure especially when interpreting and comparing results across different brain regions. Lastly, both the per-voxel permutation test and *q*-value computation assume that each test is independent of the other and do not account for the spatial correlation encountered in brain MRI scans. Brain images often exhibit spatial correlation and adjacent voxels can be influenced by similar underlying factors. Ignoring this correlation may affect the accuracy of the statistical analysis and lead to biased results. Compared to the above approach, the Perm95 test that we used exhibits notable advantages. Firstly, this method requires only 1 free parameter, specifically the selection of the extreme percentile (95% in our case) of the whole-brain test-statistic value used in computing the null distribution. In contrast, the *q*-value approach involves 2 free parameters, namely the threshold for the tumor probabilities, and the region used for *q*-value computation. The reduced parameter complexity of the Perm95 test enhances its simplicity and ease of implementation. Furthermore, the Perm95 test demonstrates robustness when confronted with deviations from the Gaussian assumptions underlying the test statistic values, allowing more reliable analysis of brain MRI data and accommodating potential non-normality in the statistical distribution. Additionally, it offers effective control over FPs by reducing family-wise error (FWE). By mitigating the occurrence of FPs, Perm95 strikes a balance between the advantages of the *q*-value based non-parametric modeling approach and the need for stringent control over FPs. In comparison to the *q*-values, the Perm95 test exhibits superior stringency as we found in our comparative analysis of the 2 techniques, providing the most optimal control over FPs.

Our results led us to interesting insights with regard to anatomical substrates associated with the various cognitive domains. Overall, left hemispheric parcels were more important across domains. Left Fronto-parietal regions have been known to be associated with A & EF, though classically these functions are believed to be bilaterally dominant.^37–39^ We also found a left-sided predominance, though right temporal regions seemed to be significant too. It may so be that A & EF is subserved by bilateral pathways connecting homologous brain regions. Though left-sided regions appear to be dominant leading to dysfunction if disrupted, certain right hemispheric nodes may also be important. Interestingly, we found that corticospinal (CST) projection fibers are significant and this corroborates a similar finding by earlier studies.^17^ Surprisingly, despite often being involved by tumor, the insula did not emerge as significant in A & EF, though earlier studies have implicated it.^17^ The language domain maps reflect our current understanding of language as a dual stream model comprising of ventral and dorsal pathways^37,40–44^ corroborating cortical and subcortical substrates known to be associated with language function. These results validate our overall technique and thereby the results for other domains also. The involvement of CST fibers and optic radiations indicate the importance of primary motor output and visual input pathways in language as assessed by our battery comprising of visual stimulus (picture naming) and a verbal response (output). With respect to memory, our results again reinforced conventional anatomical substrates known to be associated with memory.^37,41,45,46^ There was a left-sided preponderance overlapping with substrates involved in language, even though our battery included both visual (traditionally though to be right dominant) and verbal tests. In 10% of our subjects who were illiterate, the visual memory test (RCFT) could not be administered and that may have contributed to the under-representation of visual memory deficits, and by extrapolation the lack of right sided substrates in our maps. We did not assess the various components of memory separately. However, our results highlight the role of anatomical regions in the occipito-temporal lobes beyond the hippocampus which usually garners a lot of interest in neuro-oncology, with efforts targeted at preserving its function as is the case in hippocampal sparing radiotherapy techniques.^47,48^ Visuospatial function is generally believed to be subserved by right sided parietal cortical areas, especially areas of the superior parietal lobule around the intraparietal sulcus and the arcuate fibers connecting it to the IFG. Though the arcuate was identified bilaterally, left ILF and IFOF seemed to be most involved and surprisingly, parietal cortical regions were not significantly involved in our study. For visuomotor speed, the regions involved seem to suggest the primary input (left visual processing areas) and output (left motor regions) nodes along with their interconnecting associative tracts and interhemispheric activation via the callosal fibers.

Overall, the perislyvian network of white matter tracts seems to be a very important component of networks subserving many of the domain functions. It is therefore not surprising to see multidomain deficits in most gliomas where this network is involved. This corroborates recent findings indicating the primacy and central role of these white matter tracts in cognitive function.^49^ Along with the primary unimodal input and output pathways, this network appears to be the key to understanding and preserving domain function during therapy for gliomas.

### Limitations

We acknowledge that the current study does have its limitations. Our subjects had on average larger tumors and this is true for the general Indian population of brain tumors. Whereas it may limit its applicability to other populations with smaller tumors, it serves as a benchmark for Indian subjects and is invaluable for comparative associations with other populations. The NCF assessment battery was not comprehensive, and many components of individual domains were not tested. For example, working, verbal, and visual memory were not individually assessed. Some of the assessments were not quantitative (the modified picture naming test we used was only interpreted subjectively). We overcame this limitation by including only severely affected cases as abnormal. Less affected subjects (where subjectivity in interpretation would be more prevalent) were included in the control group. Besides this, in the analysis, while attributing the association of anatomical parcels to specific domain function, we must also bear in mind a few caveats. Multidomain involvement is the norm in gliomas and it could be that a parcel strongly implicated in domain can interfere with the function in another domain without necessarily directly being responsible for the function of that domain.^5^ In interpreting the relevant parcels, the false positive rate for the heatmaps was set at 20% (*P* value 0.2). We did give cognizance to *P* = .05 also, but intentionally took a lower threshold since this was an exploratory analysis and we wanted to be more sensitive in the identification of clinically relevant parcels. Finally, it is difficult to attribute a causal association between the amount (in terms of percentage) of involvement of a particular parcel and neurocognitive function. For example, even a 5% or 10% tumor involvement of a very critical parcel can lead to dysfunction, whereas more extensive involvement of less important ones can be insignificant clinically. On the other hand, using an all-or-none criterion may exclude many critical parcels. We used the top 15 involved as an indicator, but this is by no means a perfect defining criterion. Accuracy and validity of subcortical tracts identified could be suboptimal due to the effects of tumor distortion which may not be adequately captured (in the absence of anatomical tractography data) while creating the brain-maps. Finally, it has been shown that lesion aetiology can affect the neurocognitive performance and may impact the findings of lesion-symptom mapping studies.^49^ Tumor-induced plasticity and reorganization of networks could also affect the results, especially with lower grade gliomas where slow growth prior to their clinical diagnosis could provide ample time for this phenomenon. Therefore, extrapolating the results to other lesions (eg, stroke) should be done cautiously.

Despite the limitations of the study, this is 1 of the largest of its kind published and the only 1 in Indian subjects using Indian specific brain templates. It is well known that brain maps of different populations differ, not only due to anthropometric differences, but also due to differences in networks influenced by specific cultural and demographic factors as in the Chinese population.^50^ This work, therefore, is an important contribution. Not only does our study validate established and previously reported findings in other demographic populations, underlining a thread of commonality, but also provides new insights into the complex workings of brain function with potential differences across populations. Larger studies across populations would be needed to validate these findings. The findings of this investigation have significant implications in selecting and customizing tests for at-risk functions based on tumor locations. This knowledge can greatly contribute to the planning of surgical procedures and rehabilitation strategies, thereby optimizing patient outcomes in the context of brain tumor management.

## Supplementary Material

vdae020_suppl_Supplementary_Data

## Data Availability

The data are available with the authors and will be made available upon reasonable request.
