# Supplementary material for: Tumor location and neurocognitive function—Unravelling the association and identifying relevant anatomical substrates in intra-axial brain tumors
Source: Neurooncol Adv. 2024 Feb 9;6(1):vdae020. doi: 10.1093/noajnl/vdae020 (PMC10924535; doi:10.1093/noajnl/vdae020)
Supplement: vdae020_suppl_Supplementary_Data [file vdae020_suppl_supplementary_data.zip › Supplementary material S1.docx]

**Supplementary Material 1:**

**Lists of Neuropsychological assessments used in this study.**

| **Neuropsychological Domains** | **Neuropsychological Tests** |
| --- | --- |
| Handedness | 1. Edinburgh Handedness Inventory (EHI)(24) |
| Attention and Executive Function | 1. ACE-III (which includes verbal fluency)(23) 2. Counting (1-20) forwards and backwards(27) 3. Trail Making B(27,28) |
| Memory | 1. ACE-III(23) 2. Rey’s -Osterrieth Complex Figure Test (RCFT- Immediate & Delayed recall)(26) 3. Rey’s Auditory Verbal Learning Test (RAVLT- Immediate & Delayed recall)(26) 4. Counting BACKWARDS (random number sequence)(27) |
| Language | 1. ACE-III(23) 2. Picture Description** 3. Action Words** 4. Naming (modified 60) ** |
| Visuospatial and visuo-constructional abilities | 1. ACE-III(23) 2. RCFT (Copy)(26) 3. Line Bisection(30) |
| Visuomotor speed | 1. Trail Making A(27,28) |
| Psychomorbid state | 1. General Health Questionnaire- 12(31,32) (GHQ12) |

* ACE is included as it tests the various domains. However, it was used only as a screening test and for the purpose of this study, only the other specific neuropsychological tests were considered to define function of a particular domain.

** Language tests were customized and modified as per our patient population

**Interpretation for each test performed as part of the overall assessment**

| **Tests** | **Normal** | **Mild/Moderate** | **Severe/Abnormal** |
| --- | --- | --- | --- |
| **ACE-III** (Mathuranath, Nestor, Berrios, Rakowicz, & Hodges, 2000; So, et al., 2018) | 88 and above | 83-88 | Below 83, Couldn’t perform |
| **Trail Making Test (TMT):** |  | | |
| **A** (Reitan, 1958) | <40 seconds | 41-78 seconds | >78 seconds, Couldn’t Perform |
| **B** (Reitan, 1958) | <78 seconds | 78-91 seconds | >91 seconds, Couldn’t Perform |
| **Naming** | 56-60 | 45-55 | <45, couldn’t perform |
| **Action Words** | All 20 named | 11-19 | <10, couldn’t perform |
| **Picture Description** | 10 Sentences | 5-9 | <5, couldn’t perform |
| **Rey’s auditory verbal learning test (RAVLT)** (Rao, Subbakrishna, & Gopukumar, 2004; Kessel, Baumfalk, Zandvoort, Robe, & Snijders, 2017 ) | >0 SD | 0 to -2 SD | < -2SD |
| **Rey complex figure test (RCFT)** (Rao, Subbakrishna, & Gopukumar, 2004; Kessel, Baumfalk, Zandvoort, Robe, & Snijders, 2017 ) | >0 SD | 0 to -2 SD | < -2SD |

* Counting and line bisection was interpreted subjectively and not graded

References:

Kessel, E. v., Baumfalk, A. E., Zandvoort, M. J., Robe, P. A., & Snijders, T. J. (2017 ). Tumor-related neurocognitive dysfunction in patients with diffuse glioma: a systematic review of neurocognitive functioning prior to anti-tumor treatment. *Journal of Neurooncology*, 134:9–18.

Mathuranath, P., Nestor, P., Berrios, G., Rakowicz, W., & Hodges, J. (2000). A brief cognitive test battery to differentiate Alzheimer’s disease and frontotemporal dementia. *Neurology*, 55(11):1613–1620.

Rao, S. L., Subbakrishna, D., & Gopukumar, K. (2004). *NIMHANS Neuropsychological Battery - Manual.* Banglore: National Institute of Mental Health & Neurosciences.

Reitan, R. (1958). Validity of the Trail Making Test as an Indicator of Organic Brain Damage. *Perceptual and Motor Skills*, 8(3), 271–276.

So, M., Foxe, D., Kumfor, F., Murray, C., Hsieh, S., Savage, G., . . . Piguet, O. (2018). Addenbrooke's Cognitive Examination III: Psychometric Characteristics and Relations to Functional Ability in Dementia. *Journal of the International Neuropsychological Society*, 4(8):854-863. doi: 10.1017/S1355617718000541.
