# Supplementary material for: Tumor location and neurocognitive function—Unravelling the association and identifying relevant anatomical substrates in intra-axial brain tumors
Source: Neurooncol Adv. 2024 Feb 9;6(1):vdae020. doi: 10.1093/noajnl/vdae020 (PMC10924535; doi:10.1093/noajnl/vdae020)
Supplement: vdae020_suppl_Supplementary_Data [file vdae020_suppl_supplementary_data.zip › Supplementary material S10 new_clean.docx]

| **Domain** | **Cortical** | | | | **Sub-cortical** | | | |
| --- | --- | --- | --- | --- | --- | --- | --- | --- |
|  | **Permutation test (95^th^ percentile – 0.2)** | | **Permutation test (95^th^ percentile- 0.05** | | **Permutation test (95^th^ percentile – 0.2)** | | **Permutation test (95^th^ percentile – 0.05)** | |
|  | **Name (no.)** | **Percent** | **Name (no.)** | **Percent** | **Name (no.)** | **Percent** | **Name (no.)** | **Percent** |
| 1. **Attention & Executive function** | 1. L_Superior-planum temporale Temporal Gyrus (84) 2. L_central Sulcus 3. L_postcentral Gyrus (115) 4. L_posterior Fissure (89) 5. L_lateral ventricle (3) 6. R_Superior-Transverse Temporal Gyrus (156) 7. L_Precentral Gyrus 8. L_Caudate 9. R_Transverse Temporal Gyrus (197) 10. L_thalamus (7) 11. L_Superior Frontal Gyrus 12. L_subcentral Gyrus & Sulcus 13. L_Superior Temporal Gyrus 14. L_inferior Precentral Gyrus 15. **L_choroid Plexus** | 50.29  44.50  27.44  18.39  12.53  10.03  9.16  8.92  7.34  2.87  1.91  1.69  1.66  1.35  **1.18** | 1. L_Superior-planum temporale Temporal Gyrus (84) 2. L_central Sulcus 3. L_posterior Fissure (89) 4. L_postcentral Gyrus (115) 5. L_lateral ventricle (3) 6. L_Caudate 7. L_Precentral Gyrus 8. L_Superior Temporal Gyrus 9. L_Subcentral Gyrus and Sulcus 10. R_Superior-Transverse Temporal Gyrus (156) 11. L_Superior Frontal Gyrus 12. R_Transverse Temporal Gyrus (197) 13. L_Thalamus 14. **L_Hippocampus** 15. L_inferior Precentral Gyrus | 42.17  31.98  13.31  12.08  5.34  5.05  4.23  1.42  1.32  1.07  0.60  0.56  0.50  **0.14**  0.12 | 1. L_Corticospinal projection network 2. L_Arcuate 3. L_Long Perisylvian segment 4. Internal Capsule 5. Corpus Callosum 6. Fornix 7. **L_Anterior Perisylvian Segment** 8. L_Cingulum 9. **L_Posterior Perisylvian Segment** | 12.20  6.98  5.81  4.96  2.72  2.69  **2.55**  1.87  **1.83** | 1. L_Corticospinal projection network 2. Internal Capsule 3. L_Arcuate 4. L_Long Perisylvian segment 5. Corpus Callosum 6. Fornix 7. L_Cingulum | 8.73  3.73  3.06  1.73  1.06  1.05  0.22 |

| **Domain** | **Cortical** | | | | **Sub-cortical** | | | |
| --- | --- | --- | --- | --- | --- | --- | --- | --- |
|  | **Permutation test (95^th^ percentile – 0.2)** | | **Permutation test (95^th^ percentile – 0.05)** | | **Permutation test (95^th^ percentile – 0.2)** | | **Permutation test (95^th^ percentile)** | |
|  | **Name (no.)** | **Percent** | **Name (no.)** | **Percent** | **Name (no.)** | **Percent** | **Name (no.)** | **Percent** |
| **Language** | 1. L_transverse Temporal Gyrus (122) 2. L_posterior Fissure (89) 3. L_vessel 4. L_Superior planum temporale Temporal Gyrus (84) 5. L_Superior-transverse Temporal Gyrus (81) 6. L_inferior Circular Insular Gyrus (96) 7. L_Pallidum (10) 8. L_inferior Lateral Ventricle 9. L_choroid plexus (20) 10. L_superior Temporal Gyrus (121) 11. L_Insular Gyrus and superior Cental Insula (65) 12. L_Hippocampus (14) 13. L_thalamus 14. L_inferior Temporal Gyrus   15. **L_Subcentral gyrus and Sulcus** | 96.19  86.31  81.25  76.58    75.79  74.77  61.53  60.98  47.93  46.91  41.25  40.28  19.60  19.28  **17.90** | 1. L_transverse Temporal Gyrus (122) 2. L_posterior Fissure (89) 3. L_Superior-transverse Temporal Gyrus (81) 4. L_vessel 5. L_inferior Circular Insular Gyrus (96) 6. L_Superior planum temporale Temporal Gyrus (84) 7. L_choroid plexus (20) 8. L_superior Temporal Gyrus (121) 9. L_inferior Lateral Ventricle 10. L_Pallidum (10)      1. L_Insular Gyrus and superior Cental Insula (65) 2. L_Hippocampus (14) 3. L_inferior Temporal Gyrus 4. **L_lateral ventricle** 5. L_thalamus | 88.57  79.16  66.81  56.25  54.87  54.17  30.77  29.53  22.35  20.17  17.25  16.17  7.16  **5.71**  5.04 | 1. L_posterior perisylvian segment 2. L_optic radiations 3. L_inferior longitudnal fasciculus 4. L_Arcuate 5. L_long perisylvian segment 6. L_inferior Occipitofrontal Fasciculus 7. L_cortico spinal projection network 8. L_cortico ponto Cerebellum 9. L_anterior perisylvian segment 10. Internal Capsule 11. L_Uncinate 12. Anterior Commissure 13. Fornix 14. L_cingulum 15. Corpus Callosum | 100  98.89  73.81  63.52  61.22  55.94  32.37  23.97  20.41  14.67  13.02  11.67  10.63  2.49  0.42 | 1. L_posterior perisylvian segment 2. L_optic radiations 3. L_inferior longitudnal fasciculus 4. L_Arcuate 5. L_long perisylvian segment 6. L_inferior Occipitofrontal Fasciculus 7. L_cortico spinal projection network 8. L_cortico ponto Cerebellum 9. Internal Capsule 10. Fornix 11. L_Uncinate 12. L_anterior perisylvian segment 13. Anterior Commissure 14. L_cingulum 15. Corpus Callosum | 97.82  96.10  58.61  47.25  45.84  44.03  15.05  12.98  7.07  5.96  3.66  3.06  2.95  1.39  0.06 |

| **Domain** | **Cortical** | | | | **Sub-cortical** | | | |
| --- | --- | --- | --- | --- | --- | --- | --- | --- |
|  | **Permutation test (95^th^ percentile – 0.2)** | | **Permutation test (95^th^ percentile – 0.05)** | | **Permutation test (95^th^ percentile – 0.2)** | | **Permutation test (95^th^ percentile)** | |
|  | **Name (no.)** | **Percent** | **Name (no.)** | **Percent** | **Name (no.)** | **Percent** | **Name (no.)** | **Percent** |
| **Memory** | 1. L_choroid plexus (20) 2. L_Superior Planum Temporale Temporal Gyrus 3. L_transverse Temporal Gyrus (122) 4. L_posterior fissure 5. L_superior Temporal Gyrus (121) 6. L_Inferior Temporal Gyrus (120) 7. L_Anterior Occipital Gyrus (107) 8. L_Pallidum 9. L_lateral Ventricle (3) 10. L_medial and lingual Occipitotemporal Gyrus (109) 11. L_calcarine sulcus (92) 12. L_Superior Transverse Temporal Gyrus 13. L_Hippocampus (14) 14. L_lateral Occipitotemporal Gyrus (108) 15. L_Thalamus (7) | 85.21  71.62  69.25  68.79  56.17  44.76  39.53  38.04  32.39  26.59  23.46  22.98  21.11  12.37  7.94 | 1. L_choroid plexus (20) 2. L_posterior fissure 3. L_Superior Planum Temporale Temporal Gyrus 4. L_transverse Temporal Gyrus (122) 5. L_superior Temporal Gyrus (121) 6. L_lateral Ventricle (3) 7. L_Inferior Temporal Gyrus (120) 8. L_Hippocampus (14) 9. L_Superior Transverse Temporal Gyrus 10. L_medial and lingual Occipitotemporal Gyrus (109) 11. L_Pallidum 12. L_calcarine sulcus (92) 13. L_Anterior Occipital Gyrus (107) 14. L_Thalamus (7)      1. L_Lateral Occipitotemporal Gyrus | 81.07  50.74  50.65  49.66  43.31  29.94  22.17  14.15  10.83  10.39  9.31  7.69  7.57  3.14  2.28 | 1. L_posterior perisylvian segment 2. L_optic radiations 3. L_inferior longitudinal fasciculus 4. L_long perisylvian segment 5. L_inferior occipitofrontal fasciculus 6. L_arcuate 7. L_cortico spinal projection network 8. L_cortico ponto cerebellum 9. Fornix 10. L_cingulum 11. Internal Capsule 12. Corpus Callosum 13. **L_Anterior Perisylvian Segment** 14. **R_Cingulum** 15. **Anterior Commisure** | 88.42  81.89  56.80  42.86  36.34  29.67  18.79  18.63  12.58  11.06  9.96  5.64  **1.36**  **0.05**  **0.03** | 1. L_posterior perisylvian segment 2. L_optic radiations 3. L_inferior longitudinal fasciculus 4. L_long perisylvian segment 5. L_inferior occipitofrontal fasciculus 6. L_arcuate 7. Fornix 8. L_cortico spinal projection network 9. L_cortico ponto cerebellum 10. L_cingulum 11. Internal Capsule 12. Corpus Callosum | 74.24  68.80  45.75  30.30  23.44  18.84  10.19  7.72  7.63    6.84  5.01  3.89 |

| **Domain** | **Cortical** | | | | **Sub-cortical** | | | |
| --- | --- | --- | --- | --- | --- | --- | --- | --- |
|  | **Permutation test (95^th^ percentile – 0.2)** | | **Permutation test (95^th^ percentile – 0.05)** | | **Permutation test (95^th^ percentile – 0.2)** | | **Permutation test (95^th^ percentile)** | |
|  | **Name (no.)** | **Percent** | **Name (no.)** | **Percent** | **Name (no.)** | **Percent** | **Name (no.)** | **Percent** |
| **Visuospatial** | 1. R_Anterior-Vertical lateral Fissure (163) 2. R_Inferior Frontal Gyrus (175) 3. R_Inferior Precentral Gyrus (191) 4. L_choroid plexus 5. L_Subparietal Gyrus 6. L_Pericallosal Sulcus 7. L_Calcarine Sulcus 8. L_thalamus 9. L_Marginal Cingulate Sulcus 10. L_superior Temporal Gyrus 11. **L_Medial and Lingual Occipitotemporal Gyrus (109)** 12. L_Lateral Ventricle 13. L_inferior Temporal Gyrus 14. R_Inferior-Opercular Frontal Gyrus (135) 15. **L_anterior Occipital Gyrus** | 45.71  41.49  40.24  34.91  34.54  27.52  22.16  20.35  17.88  16.81  **15.85**  14.51  13.83  13.63  **11.61** | 1. L_Subparietal Gyrus 2. R_Anterior-Vertical lateral Fissure (163) 3. R_Inferior Precentral Gyrus (191) 4. R_Inferior Frontal Gyrus (175) 5. L_thalamus 6. L_Calcarine Sulcus 7. L_choroid plexus 8. L_superior Temporal Gyrus 9. L_Lateral Ventricle 10. L_Marginal Cingulate Sulcus 11. **L_Hippocampus** 12. L_Pericallosal Sulcus 13. R_Inferior-Opercular Frontal Gyrus (135) 14. L_inferior Temporal Gyrus 15. **R_Precentral Gyrus** | 16.05  15.48  13.71  13.17  12.75  9.89  9.47  7.33  4.81  4.60  **4.13**  3.76  3.61  2.51  **1.71** | 1. L_cingulum 2. L_inferior longitudinal fasciculus 3. L_inferior occipitofrontal fasciculus 4. Corpus Callosum 5. L_Posterior Perisylvian Segment 6. L_optic radiations 7. L_Arcuate 8. Fornix 9. L_Cortico_Ponto_Cerebellum 10. L_Corticospinal Projections network 11. Internal capsule 12. R_cortico spinal projections network 13. **L_long perisylvian segment** 14. **R_cingulum** 15. **L_anterior perisylvian segment** | 22.32  13.88  12.46  10.43  8.27  6.96  6.42  5.57  1.98  1.50  1.38  0.62  **0.31**  **0.18**  **0.17** | 1. L_cingulum 2. Corpus Callosum 3. L_inferior occipitofrontal fasciculus 4. L_inferior longitudinal fasciculus 5. Fornix 6. L_Arcuate 7. L_posterior perisylvian segment 8. L_optic radiations 9. L_Cortico_Ponto_Cerebellum 10. Internal capsule 11. L_Corticospinal Projections 12. R_cortico spinal projections | 12.00  4.77  2.64  1.67  1.60  1.13  0.61  0.56  0.31  0.24  0.18  0.11 |

| **Domain** | **Cortical** | | | | **Sub-cortical** | | | |
| --- | --- | --- | --- | --- | --- | --- | --- | --- |
|  | **Permutation test (95^th^ percentile – 0.2)** | | **Permutation test (95^th^ percentile – 0.05)** | | **Permutation test (95^th^ percentile – 0.2)** | | **Permutation test (95^th^ percentile)** | |
|  | **Name (no.)** | **Percent** | **Name (no.)** | **Percent** | **Name (no.)** | **Percent** | **Name (no.)** | **Percent** |
| **Visuomotor** | 1. L_Choroid Plexus (20) 2. L_Pallidum 3. L_Central Sulcus 4. L_lateral Ventricle (3) 5. L_Thalamus (7) 6. L_Caudate (8) 7. L_Hippocampus (14) 8. L_Superior Temporal Gyrus 9. L_inferior temporal Gyrus (120) 10. L_Precentral Gyrus 11. L_Inferior Lateral Ventricle 12. L_Superior Planum Temporale Temporal Gyrus 13. **L_Anterior Occipital Gyrus** 14. L_Marginal Cingulate Sulcus 15. L_Medial and Lingual Occipitotemporal Gyrus (109) | 80.47  61.74  49.56  40.02  29.76  26.15  21.35  17.94  14.36  12.26  9.09  8.76  **8.30**  8.18  8.05 | 1. L_Choroid Plexus (20) 2. L_Pallidum 3. L_Central Sulcus 4. L_lateral Ventricle (3) 5. L_Caudate (8) 6. L_Thalamus (7) 7. L_Hippocampus (14) 8. L_inferior temporal Gyrus (120) 9. L_Superior Temporal Gyrus 10. L_Marginal Cingulate Sulcus 11. **L_Postcentral Gyrus (115)** 12. L_Inferior Lateral Ventricle 13. L_Superior Planum Temporale Temporal Gyrus 14. L_Medial and Lingual Occipitotemporal Gyrus (109) 15. L_Precentral Gyrus | 60.95  46.50  32.20  25.05  14.48  12.74  10.36  7.85  7.22  3.41  **2.66**  2.27  2.23  2.16  1.92 | 1. L_optic radiations 2. L_posterior perisylvian segment 3. L_inferior longitudinal fasciculus 4. L_cortico spinal projection netwroks 5. L_inferior occipitofrontal fasciculus 6. L_long perisylvian segment 7. Internal capsule 8. L_cortico ponto cerebellum 9. L_arcuate 10. Fornix 11. L_cingulum 12. Corpus Callosum 13. **L_Anterior Perisylvian Segment** 14. Anterior Commissure 15. **R_Cortico Spinal projection network** | 78.27  51.44  49.82  41.77  34.83  22.61  17.36  17.25  17.03  16.08  8.84  7.35  **2.04**  0.45  **0.16** | 1. L_optic radiations 2. L_posterior perisylvian segment 3. L_inferior longitudinal fasciculus 4. L_cortico spinal projection network 5. L_inferior occipitofrontal fasciculus 6. L_long perisylvian segment 7. Internal capsule 8. L_cortico ponto cerebellum 9. L_arcuate 10. Fornix 11. L_cingulum 12. Corpus Callosum 13. Anterior Commissure | 59.61  43.08  42.31  31.45  26.18  16.64  13.03  11.76  9.92  7.68  3.92  3.67  0.06 |
