# Supplementary material for: Tumor location and neurocognitive function—Unravelling the association and identifying relevant anatomical substrates in intra-axial brain tumors
Source: Neurooncol Adv. 2024 Feb 9;6(1):vdae020. doi: 10.1093/noajnl/vdae020 (PMC10924535; doi:10.1093/noajnl/vdae020)
Supplement: vdae020_suppl_Supplementary_Data [file vdae020_suppl_supplementary_data.zip › Supplementary material S11 table of factors affecting NCF.docx]

Supplementary Material 11: Table shows the association of various categorical and continuous variables with neurocognitive dysfunction

|  |  | **A & EF ***  **(N = 85)** | **Memory**  **(N = 95)** | **Language**  **(N= 97)** | **Visuospatial**  **(N = 88)** | **Visuomotor**  **(N = 92)** | **Overall**  **(N = 100)** |
| --- | --- | --- | --- | --- | --- | --- | --- |
| **Categorical Variables (% of ‘affected’ are shown for each domain)** | | | | | | | |
| **Gender** | **Male** | 74.6 % | 63.8 % | 20.6 % | 46.2 % | 43.3 % | 91.4 % |
|  | **Female** | 86.4 % | 61.5 % | 34.5 % | 43.5 % | 72 % | 96.7 % |
|  | **p** | 0.25 | 0.84 | 0.15 | 0.82 | **0.01** | 0.35 |
| **Laterality** | **Left** | 82.1 % | 68.8 % | 26.5% | 41.4 % | 58.1 % | 92.8 % |
|  | **Right** | 69 % | 51.6 % | 20.7 % | 53.3 % | 36.7 % | 93.5 % |
|  | **P** | 0.17 | 0.10 | 0.55 | 0.29 | **0.05** | 1.0 |
| **Histology** | **LrGG** | 71.4 % | 59.6 % | 12.7 % | 42.6 % | 40.7 % | 93 % |
|  | **GBM** | 86.1 % | 68.4 % | 40.5 % | 50 % | 65.8 % | 93 % |
|  | **P** | 0.11 | 0.39 | **0.02** | 0.50 | **0.02** | 1.00 |
| **Continuous variables (Means for the affected group are shown with corresponding values for the control group in brackets) #** | | | | | | | |
| **T volume**  **(mean)** | **Mean** | 100.3 (91.3) | 98.1 (89.2) | 127.9 (88.7) | 113 (84.2) | 117.1 (81.8) | 98.1 (84.1) |
|  | **P** | 0.56 | 0.45 | **0.013** | **0.03** | **0.006** | 0.45 |
| **Age** | **Mean** | 42.2 (32.5) | 43.7 (38.2) | 50 (39.5) | 41.8 (37.5) | 44.4 (35.8) | 42 (37.3) |
|  | **P** | **0.003** | **0.04** | **0.004** | 0.1 | **0.001** | 0.4 |

* A & EF – Attention and Executive Function

# For continuous variables, independent t test used, and p values reported assuming unequal variances
