# Supplementary material for: Tumor location and neurocognitive function—Unravelling the association and identifying relevant anatomical substrates in intra-axial brain tumors
Source: Neurooncol Adv. 2024 Feb 9;6(1):vdae020. doi: 10.1093/noajnl/vdae020 (PMC10924535; doi:10.1093/noajnl/vdae020)
Supplement: vdae020_suppl_Supplementary_Data [file vdae020_suppl_supplementary_data.zip › Supplementary Material S12 Table LGG VS GBM.docx]

**Supplementary Material Table 12: Clinic Radiological characteristics of LGG and GBMs**

|  |  | LGG (57) | GBM (43) | p |
| --- | --- | --- | --- | --- |
| Age | Mean | 37.4 cc | 47.4 cc | < 0.001 |
|  | SD | 10.9 | 14.2 |  |
| Gender | Male | 41 | 28 | 0.63 |
|  | Female | 16 | 14 |  |
| Laterality | Left | 41 | 29 | 0.47 |
|  | Right | 16 | 15 |  |
| T volume | Mean | 88.4 cc | 108.8 cc | 0.11 |
|  | SD | 57.9 | 65.8 |  |
