# Supplementary material for: Tumor location and neurocognitive function—Unravelling the association and identifying relevant anatomical substrates in intra-axial brain tumors
Source: Neurooncol Adv. 2024 Feb 9;6(1):vdae020. doi: 10.1093/noajnl/vdae020 (PMC10924535; doi:10.1093/noajnl/vdae020)
Supplement: vdae020_suppl_Supplementary_Data [file vdae020_suppl_supplementary_data.zip › Supplementary Material S13 - LGG Maps.docx]

**LGG subgroup analysis**

Figure 1.LGG: Tumor Mean Distribution in T1 and T2 MRI Scans for LGG sub group. The figure displays the mean tumor distribution across the LGG group of patients. Higher values indicate a significant proportion of patients with tumor occurrence in that specific region of the brain.


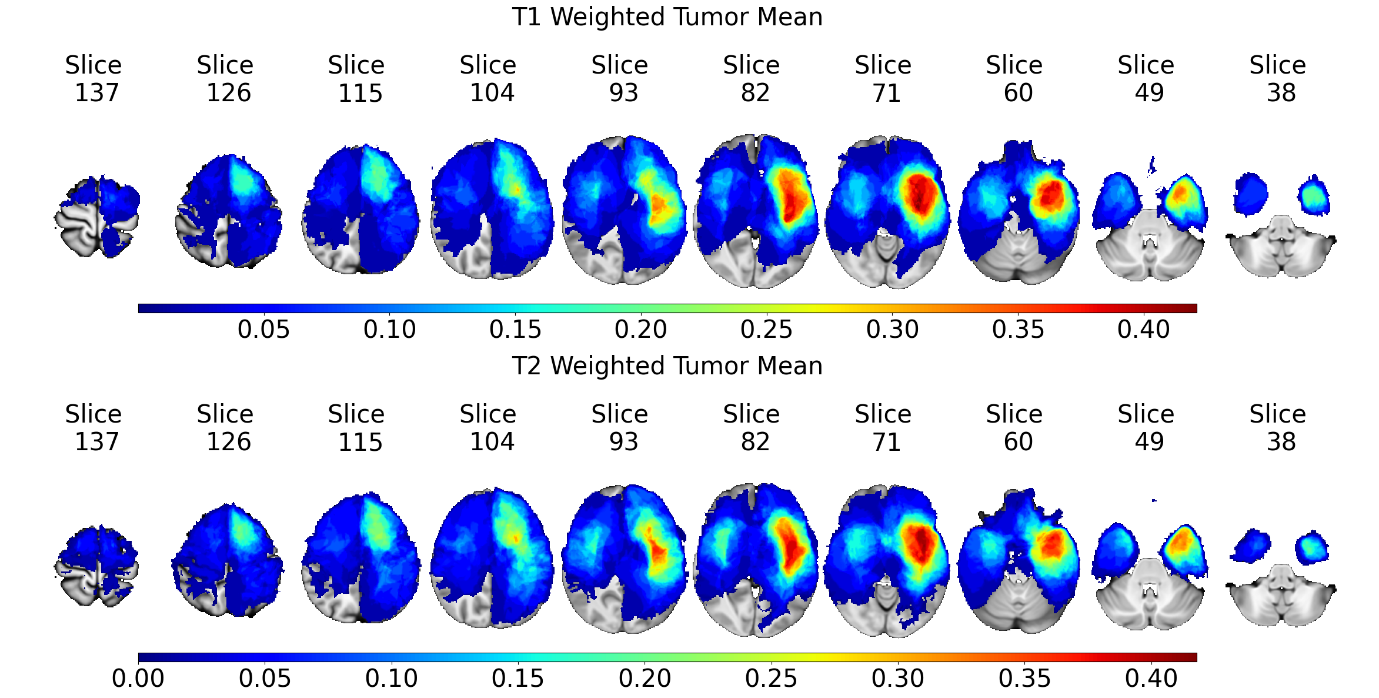


Figure 2 LGG: Perm95-Test Results for all Neurocognitive Functionality (NCF). The figure showcases brain slices representing different NCF measures, with accompanying p-values obtained from the perm95-test analysis. The output is thresholded at alpha (p) = 0.05, and only values below this threshold are displayed, highlighting brain regions with statistically significant differences in NCF between the control and affected groups.


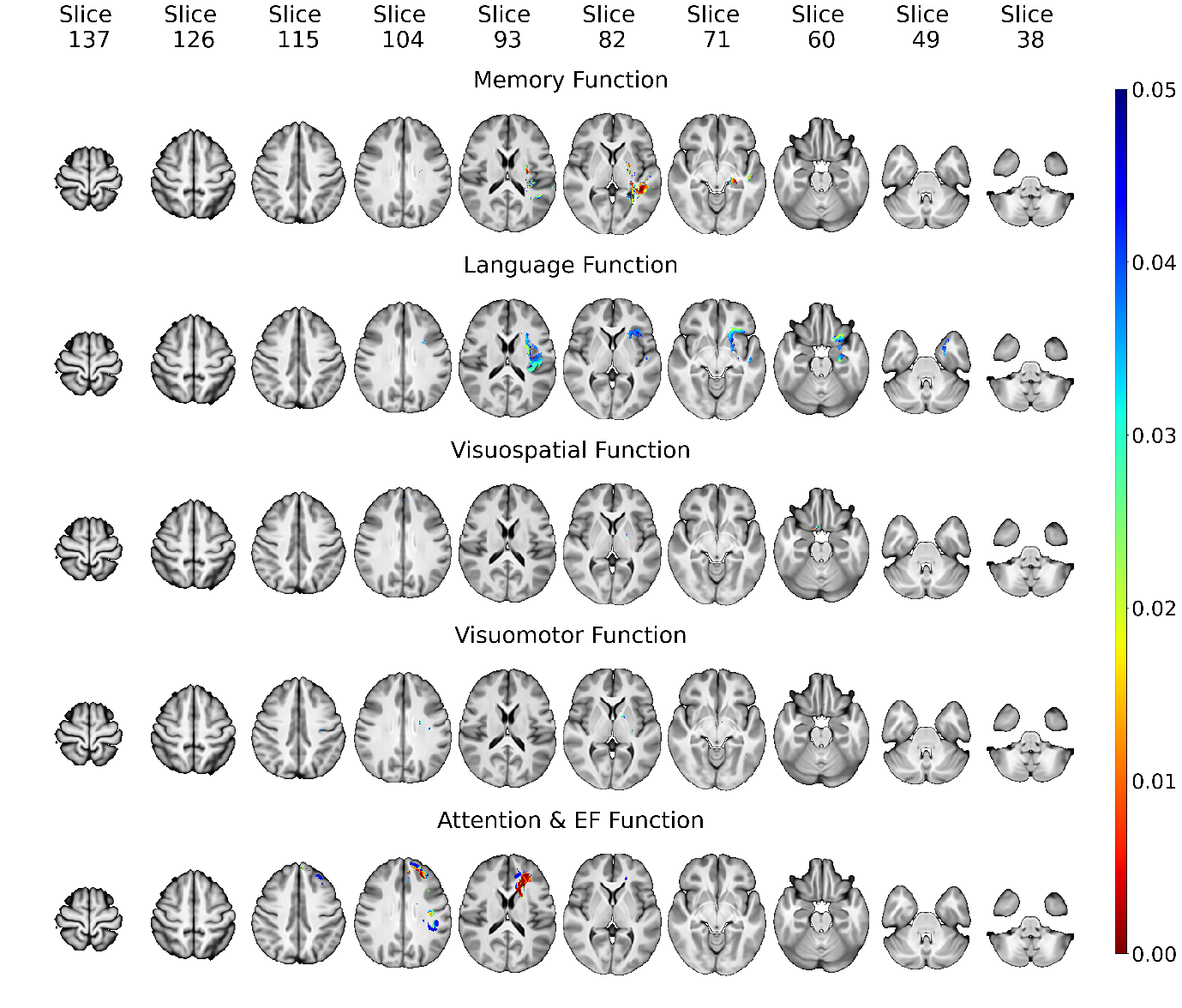


Figure 3 LGG: Heatmaps of significant cortical parcels and subcortical parcels implicated in various neurocognitive domain (x-axis) dysfunction. All subcortical parcels and top 40 cortical parcels which are predominantly covered by statistically significant voxels are shown. Each cell within the heatmap indicates the percentage of a specific cortical region that is covered by the significant voxels, obtained through a perm95-test analysis with a p-value threshold of 0. 05. The heatmaps provide a visual representation of the distribution and magnitude of statistically significant differences in NCF across the most prominently affected parcels.
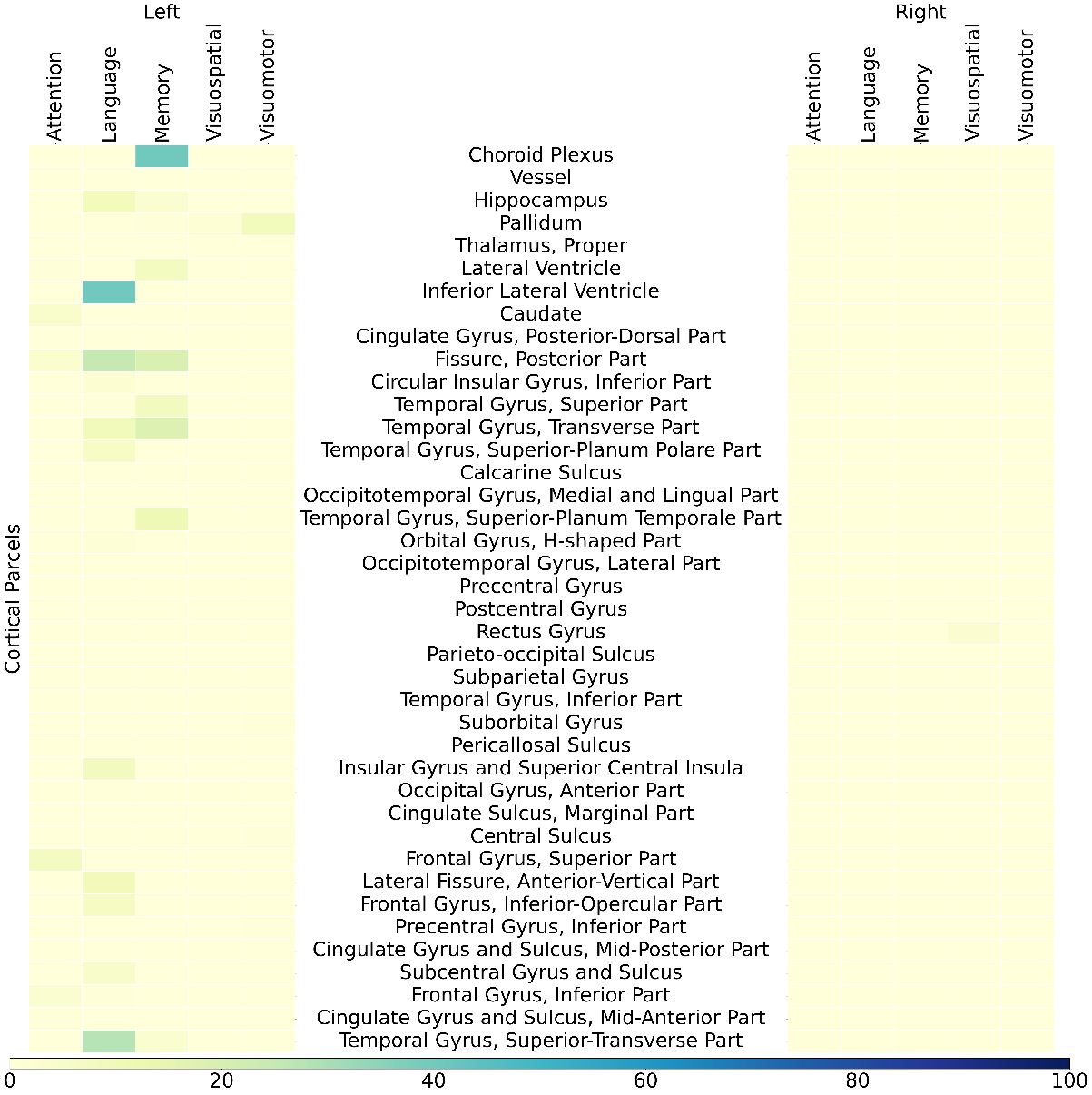


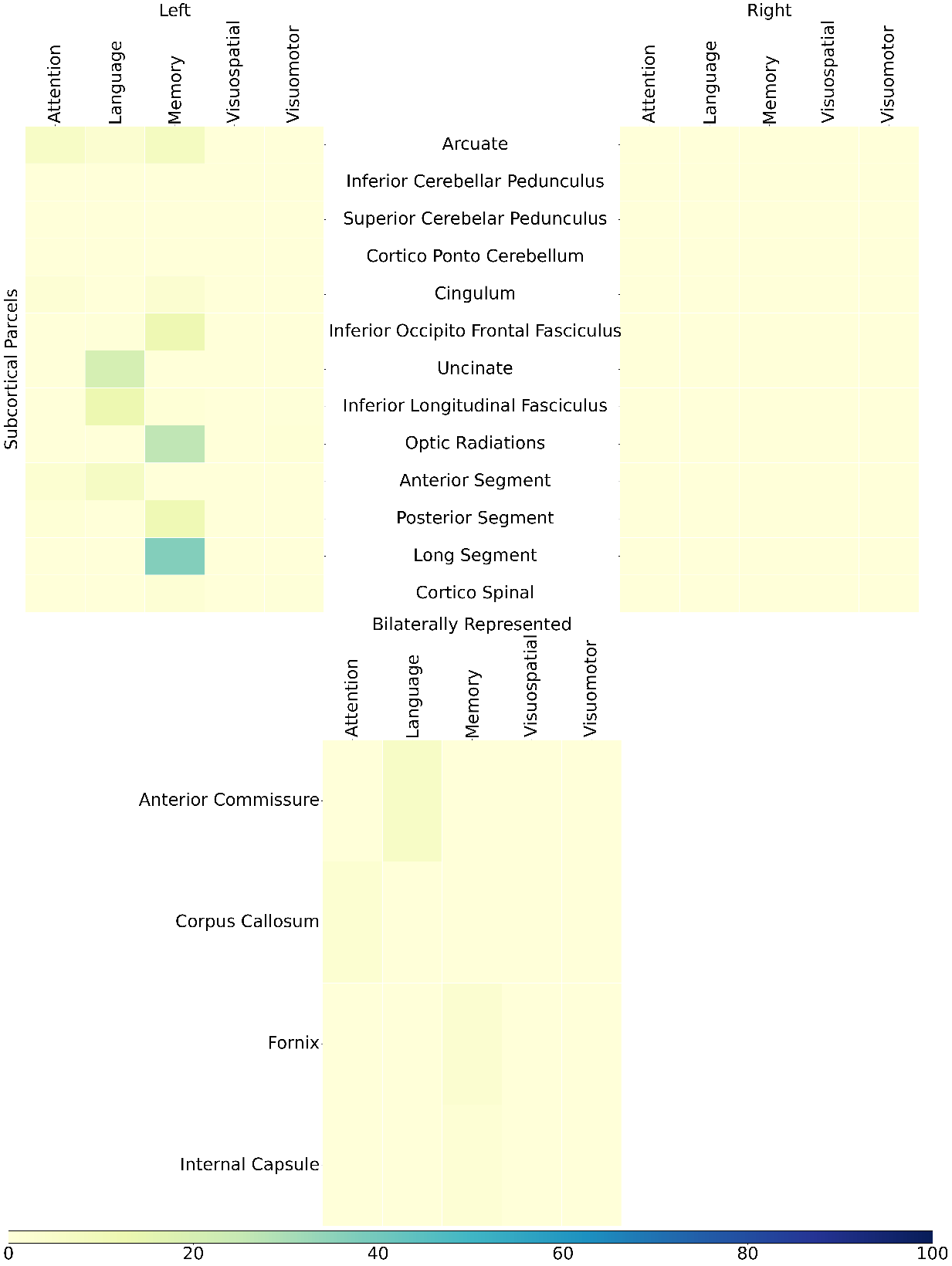


Table : Comparative list of top parcels (cortical and subcortical) based on the perm95 test for p < 0.05.

**CORTICAL**

Results for Attention

Parcel Name % of Area Covered

0 Left Frontal Gyrus, Superior Part 7.72

1 Left Caudate 4.68

2 Left Fissure, Posterior Part 3.87

3 Left Frontal Gyrus, Inferior Part 3.25

4 Right Circular Insular Gyrus, Inferior Part 0.85

5 Right Fissure, Posterior Part 0.61

6 Left Cingulate Gyrus and Sulcus, Mid-Anterior Part 0.50

7 Left Lateral Ventricle 0.43

8 Right Temporal Gyrus, Superior-Transverse Part 0.40

9 Left Central Sulcus 0.09

10 Left Precentral Gyrus, Inferior Part 0.06

11 Left Choroid Plexus 0.00

12 Left Vessel 0.00

13 Left Hippocampus 0.00

14 Left Pallidum 0.00

Results for Language

Parcel Name % of Area Covered

0 Left Inferior Lateral Ventricle 40.15

1 Left Temporal Gyrus, Superior-Transverse Part 27.90

2 Left Fissure, Posterior Part 25.20

3 Left Temporal Gyrus, Transverse Part 9.66

4 Left Lateral Fissure, Anterior-Vertical Part 9.38

5 Left Hippocampus 9.27

6 Left Insular Gyrus and Superior Central Insula 8.35

7 Left Frontal Gyrus, Inferior-Opercular Part 7.27

8 Left Temporal Gyrus, Superior-Planum Polare Part 6.17

9 Left Subcentral Gyrus and Sulcus 5.00

10 Left Circular Insular Gyrus, Inferior Part 2.53

11 Left Orbital Gyrus, H-shaped Part 1.33

12 Left Temporal Gyrus, Superior Part 0.69

13 Left Frontal Gyrus, Inferior Part 0.41

14 Left Caudate 0.22

Results for Memory

Parcel Name % of Area Covered

0 Left Choroid Plexus 40.24

1 Left Fissure, Posterior Part 18.82

2 Left Temporal Gyrus, Transverse Part 17.82

3 Left Temporal Gyrus, Superior-Planum Temporale Part 11.14

4 Left Temporal Gyrus, Superior Part 8.33

5 Left Lateral Ventricle 8.14

6 Left Temporal Gyrus, Superior-Transverse Part 3.52

7 Left Hippocampus 3.37

8 Left Pallidum 0.75

9 Left Thalamus, Proper 0.36

10 Left Temporal Gyrus, Inferior Part 0.34

11 Left Occipitotemporal Gyrus, Medial and Lingual Part 0.21

12 Left Occipitotemporal Gyrus, Lateral Part 0.13

13 Left Circular Insular Gyrus, Inferior Part 0.04

14 Left Vessel 0.00

Results for Visuospatial

Parcel Name % of Area Covered

0 Right Rectus Gyrus 2.95

1 Left Pallidum 1.61

2 Left Occipitotemporal Gyrus, Lateral Part 0.13

3 Right Cingulate Gyrus and Sulcus, Mid-Anterior Part 0.13

4 Left Occipitotemporal Gyrus, Medial and Lingual Part 0.07

5 Left Thalamus, Proper 0.01

6 Right Frontal Gyrus, Superior Part 0.01

7 Left Choroid Plexus 0.00

8 Left Vessel 0.00

9 Left Hippocampus 0.00

10 Left Lateral Ventricle 0.00

11 Left Inferior Lateral Ventricle 0.00

12 Left Caudate 0.00

13 Left Cingulate Gyrus, Posterior-Dorsal Part 0.00

14 Left Fissure, Posterior Part 0.00

Results for Visuomotor

Parcel Name % of Area Covered

0 Left Pallidum 9.36

1 Left Suborbital Gyrus 0.98

2 Left Precentral Gyrus, Inferior Part 0.68

3 Left Central Sulcus 0.55

4 Left Caudate 0.33

5 Left Rectus Gyrus 0.21

6 Left Thalamus, Proper 0.16

7 Right Rectus Gyrus 0.07

8 Left Occipitotemporal Gyrus, Lateral Part 0.04

9 Left Lateral Ventricle 0.02

10 Left Choroid Plexus 0.00

11 Left Vessel 0.00

12 Left Hippocampus 0.00

13 Left Inferior Lateral Ventricle 0.00

14 Left Cingulate Gyrus, Posterior-Dorsal Part 0.00

**SUBCORTICAL**

Results for Attention

Parcel Name % of Area Covered

0 Arcuate_Left 5.98

1 Corpus_Callosum 2.61

2 Anterior_Segment_Left 2.38

3 Cingulum_Left 1.76

4 Long_Segment_Left 1.10

5 Internal_Capsule 0.06

6 Cortico_Spinal_Left 0.03

7 Anterior_Commissure 0.00

8 Arcuate_Right 0.00

9 Inferior_Cerebellar_Pedunculus_Right 0.00

10 Superior_Cerebelar_Pedunculus_Right 0.00

11 Cortico_Ponto_Cerebellum_Right 0.00

12 Inferior_Cerebellar_Pedunculus_Left 0.00

13 Superior_Cerebelar_Pedunculus_Left 0.00

14 Cortico_Ponto_Cerebellum_Left 0.00

Results for Language

Parcel Name % of Area Covered

0 Uncinate_Left 20.67

1 Inferior_Occipito_Frontal_Fasciculus_Left 12.70

2 Anterior_Segment_Left 6.80

3 Anterior_Commissure 5.92

4 Arcuate_Left 2.99

5 Inferior_Longitudinal_Fasciculus_Left 0.58

6 Long_Segment_Left 0.16

7 Cortico_Spinal_Left 0.09

8 Fornix 0.02

9 Internal_Capsule 0.01

10 Arcuate_Right 0.00

11 Inferior_Cerebellar_Pedunculus_Right 0.00

12 Superior_Cerebelar_Pedunculus_Right 0.00

13 Cortico_Ponto_Cerebellum_Right 0.00

14 Inferior_Cerebellar_Pedunculus_Left 0.00

Results for Memory

Parcel Name % of Area Covered

0 Posterior_Segment_Left 36.73

1 Optic_Radiations_Left 26.18

2 Inferior_Longitudinal_Fasciculus_Left 11.78

3 Long_Segment_Left 11.62

4 Arcuate_Left 7.69

5 Fornix 2.86

6 Cingulum_Left 2.75

7 Cortico_Spinal_Left 1.83

8 Internal_Capsule 1.72

9 Inferior_Occipito_Frontal_Fasciculus_Left 1.06

10 Corpus_Callosum 0.34

11 Cortico_Ponto_Cerebellum_Left 0.31

12 Anterior_Commissure 0.00

13 Arcuate_Right 0.00

14 Inferior_Cerebellar_Pedunculus_Right 0.00

Results for Visuospatial

Parcel Name % of Area Covered

0 Cortico_Ponto_Cerebellum_Left 0.31

1 Cortico_Spinal_Right 0.11

2 Cortico_Spinal_Left 0.07

3 Internal_Capsule 0.04

4 Cingulum_Left 0.02

5 Corpus_Callosum 0.02

6 Anterior_Commissure 0.00

7 Arcuate_Left 0.00

8 Arcuate_Right 0.00

9 Inferior_Cerebellar_Pedunculus_Right 0.00

10 Superior_Cerebelar_Pedunculus_Right 0.00

11 Cortico_Ponto_Cerebellum_Right 0.00

12 Inferior_Cerebellar_Pedunculus_Left 0.00

13 Superior_Cerebelar_Pedunculus_Left 0.00

14 Cingulum_Right 0.00

Results for Visuomotor

Parcel Name % of Area Covered

0 Optic_Radiations_Left 1.11

1 Inferior_Occipito_Frontal_Fasciculus_Left 0.65

2 Cortico_Spinal_Left 0.47

3 Inferior_Longitudinal_Fasciculus_Left 0.15

4 Fornix 0.14

5 Anterior_Commissure 0.06

6 Internal_Capsule 0.06

7 Arcuate_Left 0.00

8 Arcuate_Right 0.00

9 Inferior_Cerebellar_Pedunculus_Right 0.00

10 Superior_Cerebelar_Pedunculus_Right 0.00

11 Cortico_Ponto_Cerebellum_Right 0.00

12 Inferior_Cerebellar_Pedunculus_Left 0.00

13 Superior_Cerebelar_Pedunculus_Left 0.00

14 Cortico_Ponto_Cerebellum_Left 0.00
