# Supplementary material for: Tumor location and neurocognitive function—Unravelling the association and identifying relevant anatomical substrates in intra-axial brain tumors
Source: Neurooncol Adv. 2024 Feb 9;6(1):vdae020. doi: 10.1093/noajnl/vdae020 (PMC10924535; doi:10.1093/noajnl/vdae020)
Supplement: vdae020_suppl_Supplementary_Data [file vdae020_suppl_supplementary_data.zip › Supplementary Material S14 - GBM Maps.docx]

**GBM subgroup analysis**

Figure 1.GBM: Tumor Mean Distribution in T1 and T2 MRI Scans of the GBMs. The figure displays the mean tumor distribution across the GBM group of patients. Higher values indicate a significant proportion of patients with tumor occurrence in that specific region of the brain.


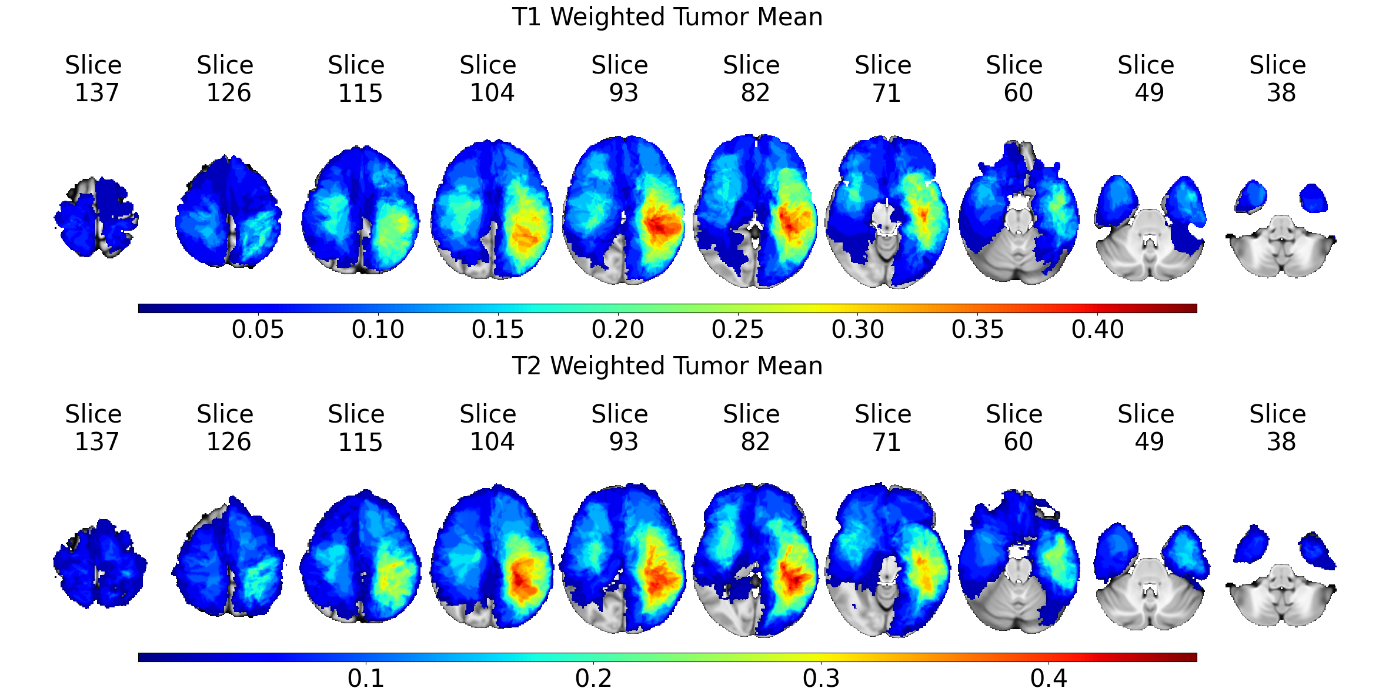


Figure 2 GBM: Perm95-Test Results for all Neurocognitive Functionality (NCF). The figure showcases brain slices representing different NCF measures, with accompanying p-values obtained from the perm95-test analysis. The output is thresholded at alpha (p) = 0. 05, and only values below this threshold are displayed, highlighting brain regions with statistically significant differences in NCF between the control and affected groups.


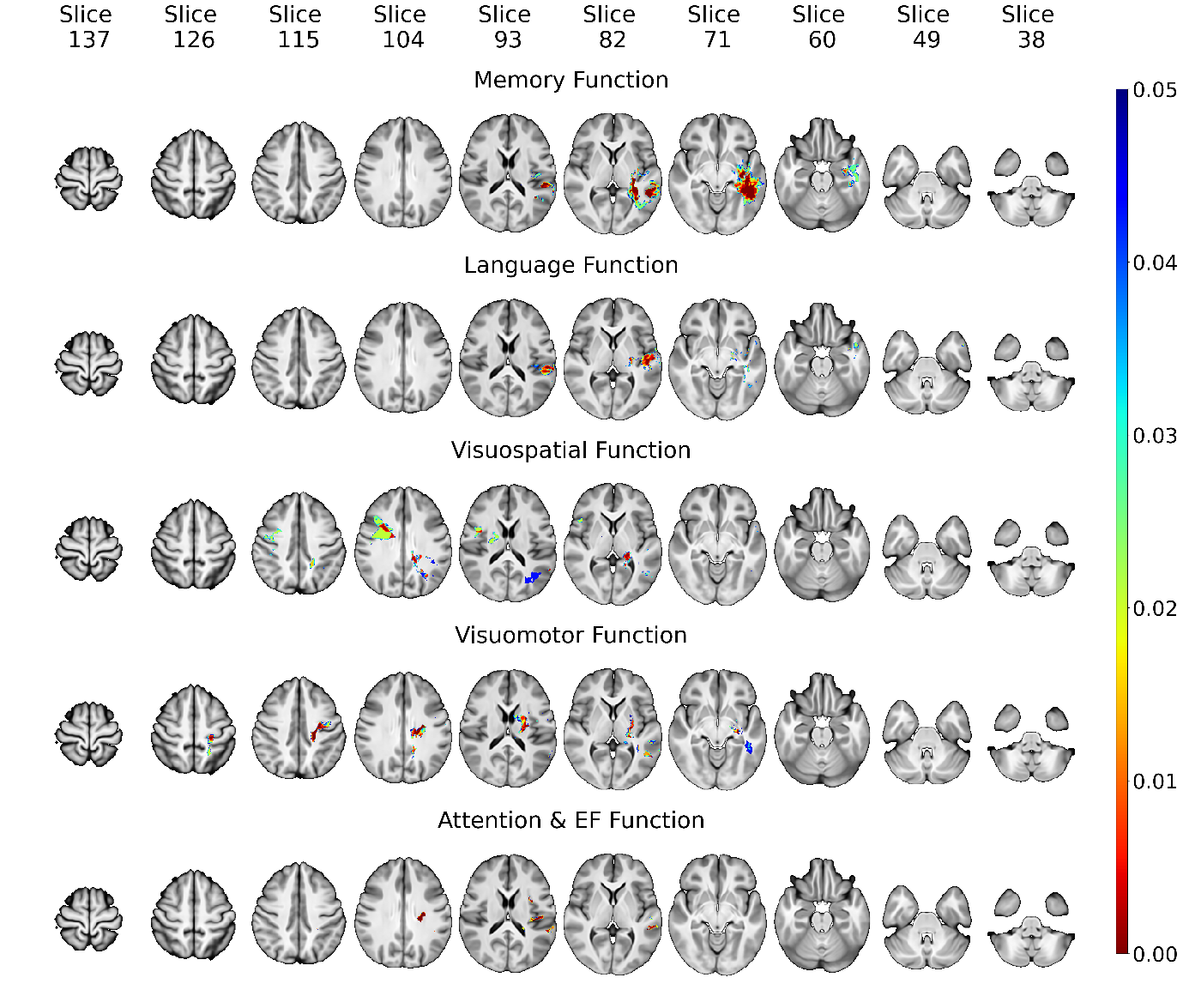


Figure 3 LGG: Heatmaps of significant cortical parcels (left side of figure) and subcortical parcels (right half of figure) implicated in various neurocognitive domain (x-axis) dysfunction. All subcortical parcels and top 40 cortical parcels which are predominantly covered by statistically significant voxels are shown. Each cell within the heatmap indicates the percentage of a specific cortical region that is covered by the significant voxels, obtained through a perm95-test analysis with a p-value threshold of 0. 05. The heatmaps provide a visual representation of the distribution and magnitude of statistically significant differences in NCF across the most prominently affected parcels.


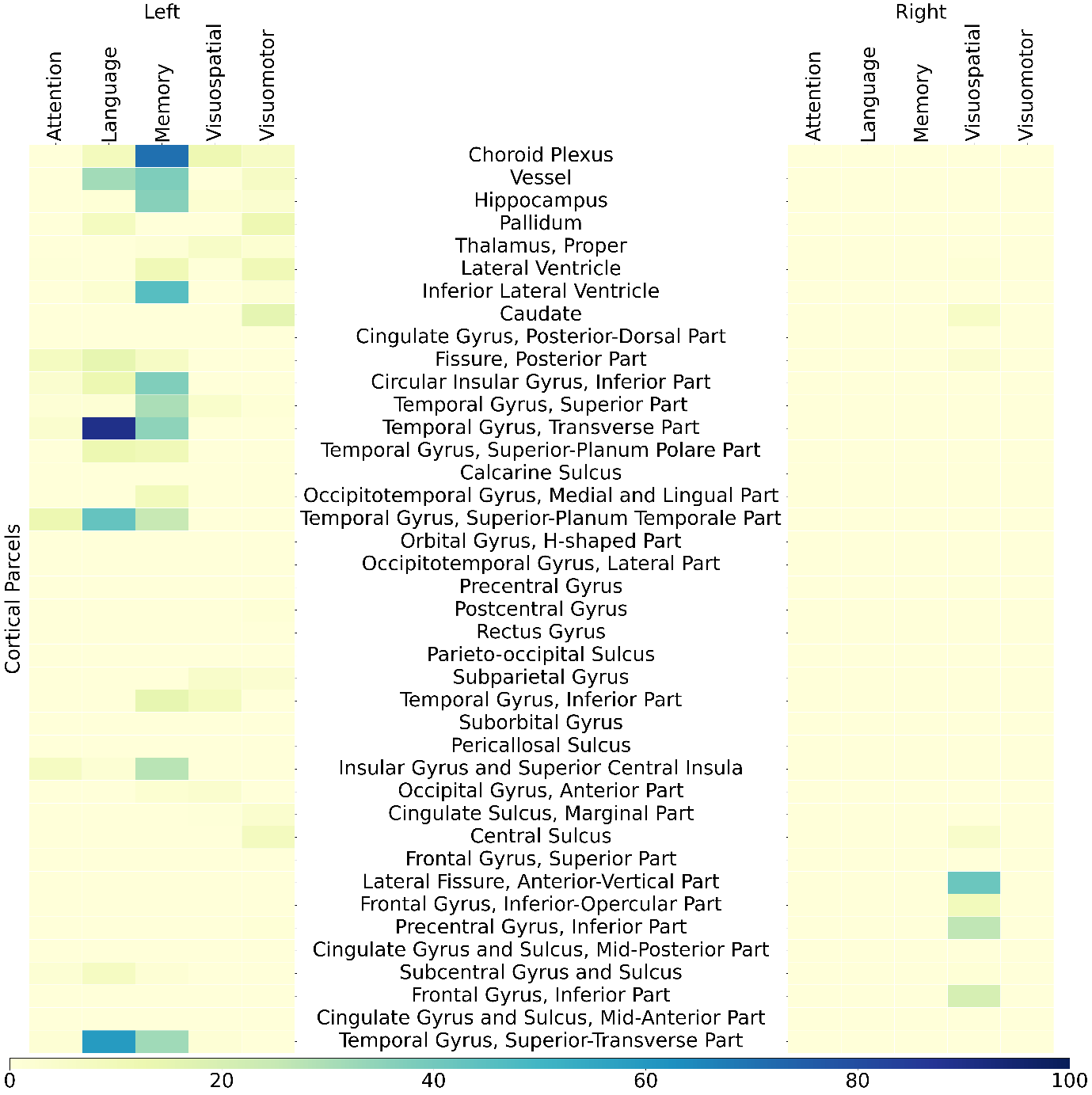


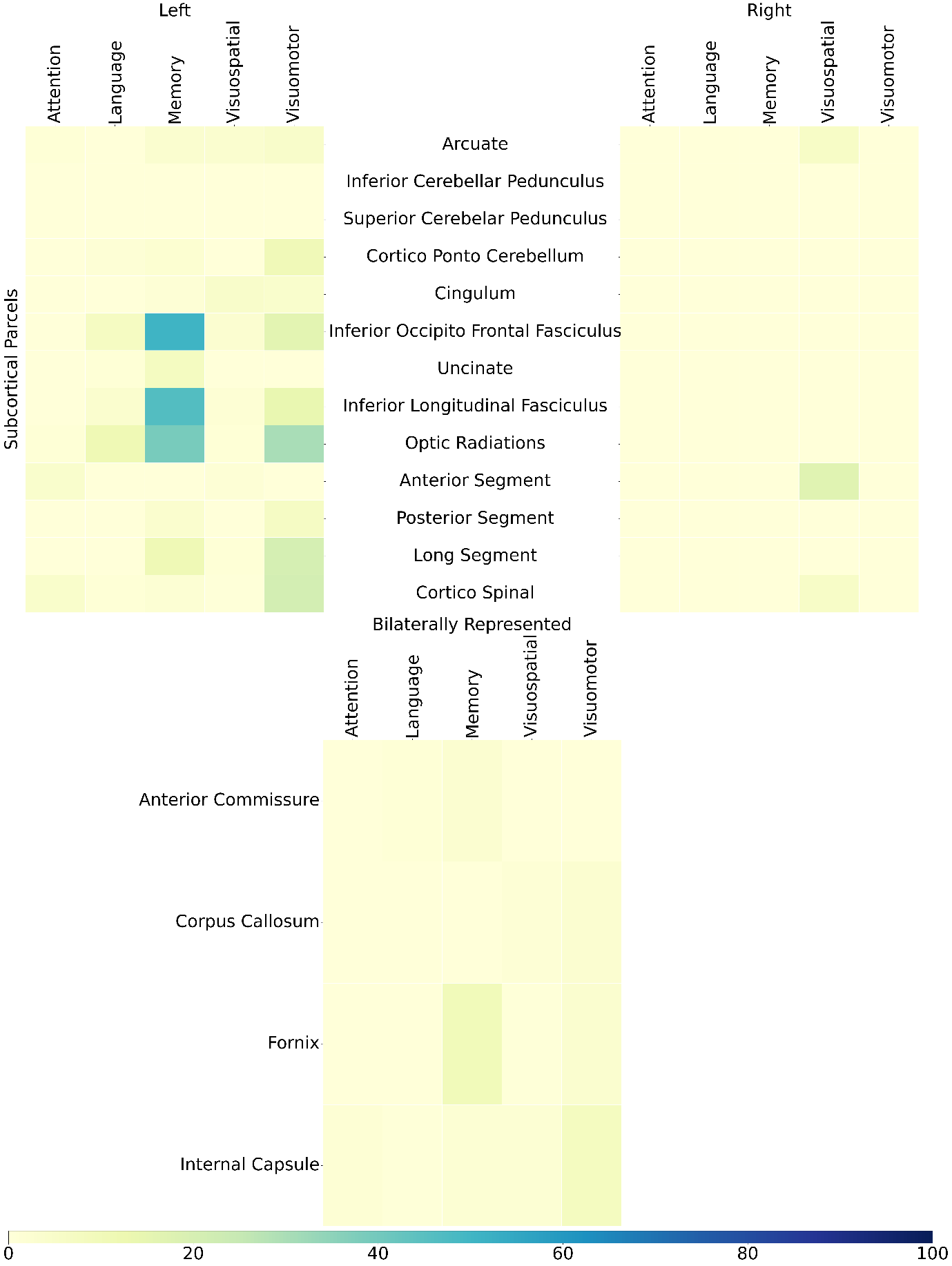


Table : Comparative list of top parcels (cortical and subcortical) based on the perm95 test for p < 0.05.

**CORTICAL**

Results for Attention

Parcel Name % of Area Covered

0 Left Temporal Gyrus, Superior-Planum Temporale Part 12.14

1 Left Fissure, Posterior Part 7.80

2 Left Insular Gyrus and Superior Central Insula 7.16

3 Left Temporal Gyrus, Transverse Part 3.54

4 Left Circular Insular Gyrus, Inferior Part 3.36

5 Left Subcentral Gyrus and Sulcus 2.25

6 Left Temporal Gyrus, Superior-Transverse Part 1.58

7 Left Temporal Gyrus, Superior Part 1.36

8 Left Lateral Ventricle 0.69

9 Left Frontal Gyrus, Inferior-Opercular Part 0.20

10 Left Postcentral Gyrus 0.09

11 Left Central Sulcus 0.07

12 Left Choroid Plexus 0.00

13 Left Vessel 0.00

14 Left Hippocampus 0.00

Results for Language

Parcel Name % of Area Covered

0 Left Temporal Gyrus, Transverse Part 89.80

1 Left Temporal Gyrus, Superior-Transverse Part 59.07

2 Left Temporal Gyrus, Superior-Planum Temporale Part 42.82

3 Left Vessel 31.25

4 Left Fissure, Posterior Part 14.84

5 Left Temporal Gyrus, Superior-Planum Polare Part 12.35

6 Left Circular Insular Gyrus, Inferior Part 12.31

7 Left Choroid Plexus 8.88

8 Left Pallidum 8.19

9 Left Subcentral Gyrus and Sulcus 7.28

10 Left Inferior Lateral Ventricle 2.65

11 Left Insular Gyrus and Superior Central Insula 2.34

12 Left Temporal Gyrus, Superior Part 1.79

13 Left Hippocampus 1.05

14 Left Thalamus, Proper 0.22

Results for Memory

Parcel Name % of Area Covered

0 Left Choroid Plexus 70.41

1 Left Inferior Lateral Ventricle 45.45

2 Left Vessel 37.50

3 Left Circular Insular Gyrus, Inferior Part 37.19

4 Left Hippocampus 36.25

5 Left Temporal Gyrus, Transverse Part 34.97

6 Left Temporal Gyrus, Superior-Transverse Part 31.87

7 Left Temporal Gyrus, Superior Part 30.05

8 Left Insular Gyrus and Superior Central Insula 27.53

9 Left Temporal Gyrus, Superior-Planum Temporale Part 24.93

10 Left Temporal Gyrus, Inferior Part 14.78

11 Left Lateral Ventricle 10.67

12 Left Temporal Gyrus, Superior-Planum Polare Part 10.35

13 Left Occipitotemporal Gyrus, Medial and Lingual Part 9.07

14 Left Fissure, Posterior Part 6.33

Results for Visuospatial

Parcel Name % of Area Covered

0 Right Lateral Fissure, Anterior-Vertical Part 41.43

1 Right Precentral Gyrus, Inferior Part 26.42

2 Right Frontal Gyrus, Inferior Part 20.11

3 Left Choroid Plexus 11.83

4 Right Frontal Gyrus, Inferior-Opercular Part 9.26

5 Left Temporal Gyrus, Inferior Part 8.08

6 Right Caudate 5.94

7 Left Thalamus, Proper 5.62

8 Right Central Sulcus 4.90

9 Left Subparietal Gyrus 4.70

10 Left Temporal Gyrus, Superior Part 4.33

11 Left Occipital Gyrus, Anterior Part 3.67

12 Right Fissure, Posterior Part 3.07

13 Left Hippocampus 2.70

14 Left Temporal Gyrus, Superior-Transverse Part 1.41

Results for Visuomotor

Parcel Name % of Area Covered

0 Left Caudate 15.29

1 Left Pallidum 11.88

2 Left Lateral Ventricle 10.90

3 Left Central Sulcus 8.26

4 Left Choroid Plexus 6.51

5 Left Vessel 6.25

6 Left Cingulate Sulcus, Marginal Part 3.90

7 Left Hippocampus 3.45

8 Left Subparietal Gyrus 3.03

9 Left Thalamus, Proper 2.43

10 Left Inferior Lateral Ventricle 1.89

11 Left Temporal Gyrus, Superior Part 1.16

12 Left Precentral Gyrus, Inferior Part 1.15

13 Left Postcentral Gyrus 0.81

14 Left Temporal Gyrus, Inferior Part 0.38

**SUBCORTICAL**

Results for Attention

Parcel Name % of Area Covered

0 Cortico_Spinal_Left 4.40

1 Anterior_Segment_Left 3.91

2 Internal_Capsule 1.88

3 Optic_Radiations_Left 0.84

4 Arcuate_Left 0.81

5 Fornix 0.21

6 Corpus_Callosum 0.04

7 Inferior_Longitudinal_Fasciculus_Left 0.02

8 Anterior_Commissure 0.00

9 Arcuate_Right 0.00

10 Inferior_Cerebellar_Pedunculus_Right 0.00

11 Superior_Cerebelar_Pedunculus_Right 0.00

12 Cortico_Ponto_Cerebellum_Right 0.00

13 Inferior_Cerebellar_Pedunculus_Left 0.00

14 Superior_Cerebelar_Pedunculus_Left 0.00

Results for Language

Parcel Name % of Area Covered

0 Optic_Radiations_Left 11.70

1 Inferior_Longitudinal_Fasciculus_Left 7.20

2 Inferior_Occipito_Frontal_Fasciculus_Left 3.81

3 Cortico_Ponto_Cerebellum_Left 1.37

4 Anterior_Commissure 1.05

5 Cortico_Spinal_Left 0.94

6 Uncinate_Left 0.83

7 Internal_Capsule 0.49

8 Arcuate_Left 0.32

9 Fornix 0.21

10 Arcuate_Right 0.00

11 Inferior_Cerebellar_Pedunculus_Right 0.00

12 Superior_Cerebelar_Pedunculus_Right 0.00

13 Cortico_Ponto_Cerebellum_Right 0.00

14 Inferior_Cerebellar_Pedunculus_Left 0.00

Results for Memory

Parcel Name % of Area Covered

0 Inferior_Longitudinal_Fasciculus_Left 50.42

1 Inferior_Occipito_Frontal_Fasciculus_Left 46.29

2 Optic_Radiations_Left 38.72

3 Posterior_Segment_Left 11.31

4 Fornix 9.90

5 Uncinate_Left 7.60

6 Long_Segment_Left 3.61

7 Arcuate_Left 3.38

8 Anterior_Commissure 3.00

9 Cortico_Ponto_Cerebellum_Left 2.44

10 Internal_Capsule 2.04

11 Cortico_Spinal_Left 2.03

12 Cingulum_Left 1.47

13 Corpus_Callosum 0.11

14 Arcuate_Right 0.00

Results for Visuospatial

Parcel Name % of Area Covered

0 Anterior_Segment_Right 16.86

1 Arcuate_Right 6.13

2 Cortico_Spinal_Right 5.81

3 Cingulum_Left 4.89

4 Arcuate_Left 3.14

5 Inferior_Longitudinal_Fasciculus_Left 3.03

6 Internal_Capsule 2.08

7 Inferior_Occipito_Frontal_Fasciculus_Left 1.82

8 Anterior_Segment_Left 1.53

9 Corpus_Callosum 1.52

10 Optic_Radiations_Left 1.11

11 Posterior_Segment_Left 0.96

12 Fornix 0.63

13 Cortico_Spinal_Left 0.40

14 Cingulum_Right 0.03

Results for Visuomotor

Parcel Name % of Area Covered

0 Optic_Radiations_Left 30.36

1 Cortico_Spinal_Left 21.47

2 Posterior_Segment_Left 20.45

3 Inferior_Longitudinal_Fasciculus_Left 15.99

4 Inferior_Occipito_Frontal_Fasciculus_Left 13.69

5 Cortico_Ponto_Cerebellum_Left 10.23

6 Internal_Capsule 7.99

7 Long_Segment_Left 6.91

8 Arcuate_Left 4.97

9 Cingulum_Left 4.18

10 Fornix 3.26

11 Corpus_Callosum 3.05

12 Uncinate_Left 0.19

13 Anterior_Commissure 0.08

14 Arcuate_Right 0.00
