# Supplementary material for: Tumor location and neurocognitive function—Unravelling the association and identifying relevant anatomical substrates in intra-axial brain tumors
Source: Neurooncol Adv. 2024 Feb 9;6(1):vdae020. doi: 10.1093/noajnl/vdae020 (PMC10924535; doi:10.1093/noajnl/vdae020)
Supplement: vdae020_suppl_Supplementary_Data [file vdae020_suppl_supplementary_data.zip › Supplementary material S2 new.docx]

**Table 2: Demographic and clinical and characteristics of the study population**

| **Variables** | | **Frequency (%)** |
| --- | --- | --- |
| **Age (years)** [Median, IQR] | | 40.50 [32.0 – 50.75] |
| **Gender** | Male | 70 (70.0) |
|  | Female | 30 (30.0) |
| **Education category** | Illiterate | 9 (9.0) |
|  | School educated | 41 (41.0) |
|  | College educated | 50 (50.0) |
| **Handedness** | Right | 98 (98.0) |
|  | Left | 1 (1.0) |
|  | Ambidextrous | 1 (1.0) |
| **Type of Surgery** | Awake | 59 (59.0) |
|  | GA | 41 (41.0) |
| **Prior Treatment** | Yes | 23 (23.0) |
|  | No | 77 (77.0) |
| **Pre-op neurodeficits** | Speech | 11 (11.0) |
|  | Motor | 12 (12.0) |
|  | Both | 8 (8.0) |
|  | None | 71 (71.0) |
| **Tumor Laterality** | Left | 69 (69.0) |
|  | Right | 31 (31.0) |
| **Lobes involved** | Frontal | 30 (30.0) |
|  | Temporal | 13 (13.0) |
|  | Parietal | 13 (13.0) |
|  | Multilobar (excluding insular tumors) | 8 (8.0) |
|  |  | Fronto-temporal 2 (2.0) |
|  |  | Temporo-parietal 1 (1.0) |
|  |  | Fronto-parietal 3 (3.0) |
|  |  | Parieto-occipital 1 (1.0) |
|  |  | Temporoparieto-occipital 1 (1.0) |
|  | Insular | 37 (37.0) |
|  |  | Pure Insular 1 (1.0) |
|  |  | Fronto-insular 4 (4.0) |
|  |  | Temporo-insular 6 (6.0) |
|  |  | Fronto-temporo insular 21 (21.0) |
|  |  | Temporo-parietal insular 2 (2.0) |
|  |  | Frontoparietal insular 1 (1) |
|  |  | Frontotemporoparietal insular 1 (1) |
| **Tumor volume (cc)** [Median, IQR] | | 90.79 [46.78 – 139.17] |
| **Histopathology** | LrGG | 57 (57.0) |
|  | GBM | 43 (43.0) |
| **IDH status** | Positive | 60 (61.2) |
|  | Negative | 38 (38.8) |

*GA – General Anaesthesia; LrGG – Lower grade (Grade 2/3) diffuse gliomas
