# Supplementary material for: Tumor location and neurocognitive function—Unravelling the association and identifying relevant anatomical substrates in intra-axial brain tumors
Source: Neurooncol Adv. 2024 Feb 9;6(1):vdae020. doi: 10.1093/noajnl/vdae020 (PMC10924535; doi:10.1093/noajnl/vdae020)
Supplement: vdae020_suppl_Supplementary_Data [file vdae020_suppl_supplementary_data.zip › Supplementary material S3.docx]

**Supplementary Material 2: Protocol for the MRI acquisition**

Equipment:

• Phillips Ingenia-70339 1.5T MRI Scanner

Brain tumor protocol:

1. T2: TR: 5400ms, TE: 107ms

2. T1W FLAIR: TR: 1600ms, TE:20ms

3. Susceptibility weighted imaging (SWI): TR: 52ms TE: 12ms

4. Diffusion weighted imaging (DWI): TR: 3000ms TE: 89ms

5. T1 PC: TR: 475ms TE: 21ms

6. T2 FLAIR: TR: 8500ms TE: 120ms

7. FSPGR: TE: 3ms TR: 8ms

8. Dynamic susceptibility perfusion contrast (DSC)

9. Magnetic resonance spectroscopy (MRS)Volumetric T2 sequence (for Neuronavigation): T2: TR: 3000ms TE: 180ms

*TR: Time to repeat

*TE: Time to echo
