# Supplementary material for: Tumor location and neurocognitive function—Unravelling the association and identifying relevant anatomical substrates in intra-axial brain tumors
Source: Neurooncol Adv. 2024 Feb 9;6(1):vdae020. doi: 10.1093/noajnl/vdae020 (PMC10924535; doi:10.1093/noajnl/vdae020)
Supplement: vdae020_suppl_Supplementary_Data [file vdae020_suppl_supplementary_data.zip › Supplementry material List with legends.docx]

Supplementary Material 1: Neurocognitive test battery used along with the interpretation for each test performed as part of the overall assessment

Supplementary Material 2: Demographic and clinical and characteristics of the study population

Supplementary Material 3: Protocol for the MRI acquisition

Supplementary Material 4: Perm95-Test Results for all Neurocognitive Functionality (NCF). The figure showcases brain slices representing different NCF measures, with accompanying p-values obtained from the perm95-test analysis. The output is thresholded at alpha (p) = 0.05, and only values below this threshold are displayed, highlighting brain regions with statistically significant differences in NCF between the control and affected groups.

Supplementary Material 5: T test methodology. One-Sided T-Test to Compare Group Means. The test follows a one-sided permutation approach, with the null hypothesis that the mean of the Control group is greater than the mean of the Affected group. The figure showcases the step-by-step process, including the calculation of mean and standard deviation for each group, the utilization of the one-sided t-statistic formula with pooled variance, and the conversion of the t-statistic to a p-value, following the one-sided t-test methodology.

Supplementary Material 6: T-Test Results for all Neurocognitive Functionality (NCF). The figure showcases brain slices representing different NCF measures, with accompanying p-values obtained from the t-test analysis. The output is thresholded at alpha (p) = 0.2, and only values below this threshold are displayed, highlighting brain regions with statistically significant differences in NCF between the control and affected groups.

Supplementary Material 7: P-Values for Per-Voxel Permutation-Test Results for all neurocognitive functionality (NCF) using relative risk as the test statistic. The figure shows brain slices representing different NCF measures, with accompanying p-values obtained from the per-voxel permutation-test analysis. The output is thresholded at alpha (p) = 0.05, and only values below this threshold are displayed, highlighting brain regions with statistically significant differences in NCF between the control and affected groups.

Supplementary Material 8: Q-Values for Per-Voxel Permutation-Test Results for all Neurocognitive Functionality (NCF) using relative risk as the test statistic. The figure shows brain slices representing different NCF measures, with accompanying q-values which are derived by adjusting the p-values obtained using per-voxel permutation-test analysis to control false positive (FP). The output is thresholded at alpha = 0.4, and only values below this threshold are displayed, highlighting brain regions with statistically significant differences in NCF between the control and affected groups.

Supplementary Material 9: Heatmaps of significant cortical parcels (left side of figure) and subcortical parcels (right half of figure) implicated in various neurocognitive domain (x-axis) dysfunction. All subcortical parcels and top 40 cortical parcels which are predominantly covered by statistically significant voxels are shown. Each cell within the heatmap indicates the percentage of a specific cortical region that is covered by the significant voxels, obtained through a perm95-test analysis with a p-value threshold of 0.05. The heatmaps provide a visual representation of the distribution and magnitude of statistically significant differences in NCF across the most prominently affected parcels.

Supplementary Material 10: Comparative list of top 15 parcels based on the perm95 test for both p < 0.2 and p < 0.05. Bold highlighted parcels indicate those with discrepancy between 0.2 and 0.05 maps.

Supplementary Material 11: Analysis of factors affecting Neurocognitive dysfunction.

Supplementary Material 12: Comparative clinic-demographic features of LGG and GBMs

Suppl Material 13 - Analysis of TLMs and probability maps of LGG

Suppl Material 14 - Analysis of TLMs and probability maps of GBM
