# Supplementary figures and images for: Tumor location and neurocognitive function—Unravelling the association and identifying relevant anatomical substrates in intra-axial brain tumors
Source: Neurooncol Adv. 2024 Feb 9;6(1):vdae020. doi: 10.1093/noajnl/vdae020 (PMC10924535; doi:10.1093/noajnl/vdae020)

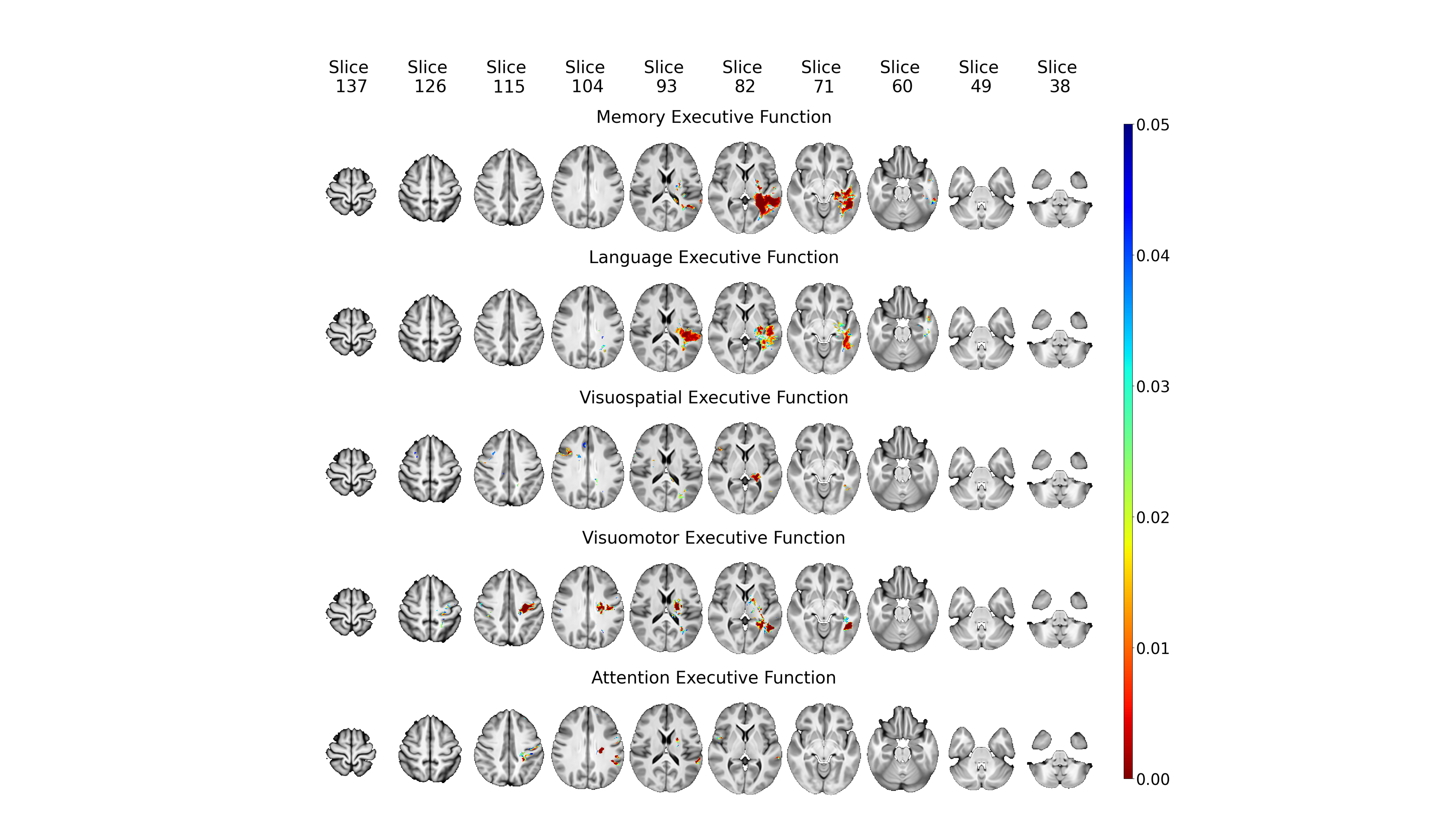

Supplement: vdae020_suppl_Supplementary_Data [file vdae020_suppl_supplementary_data.zip › Supplementary Material S4.tif]

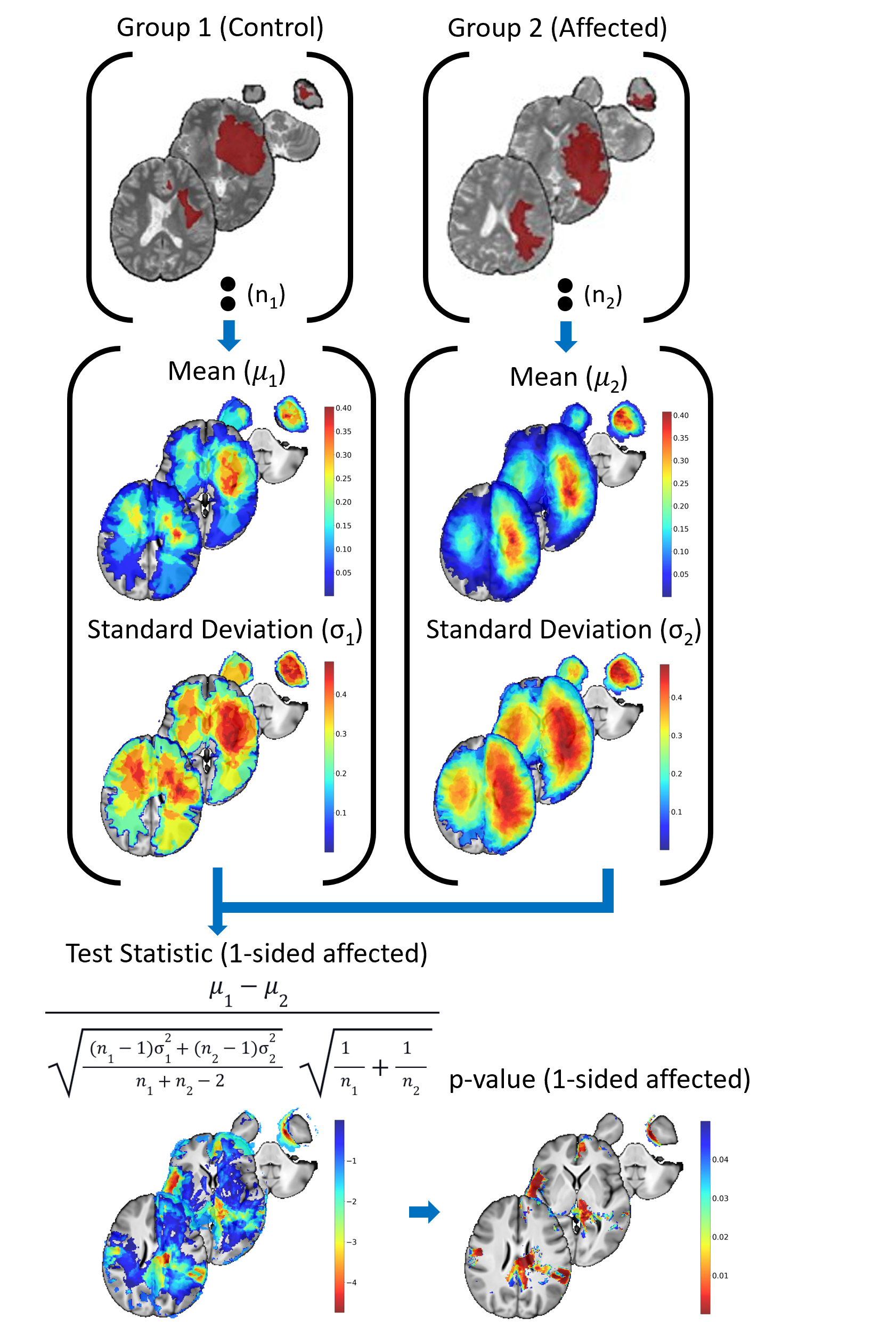

Supplement: vdae020_suppl_Supplementary_Data [file vdae020_suppl_supplementary_data.zip › Supplementary Material S5.tif]

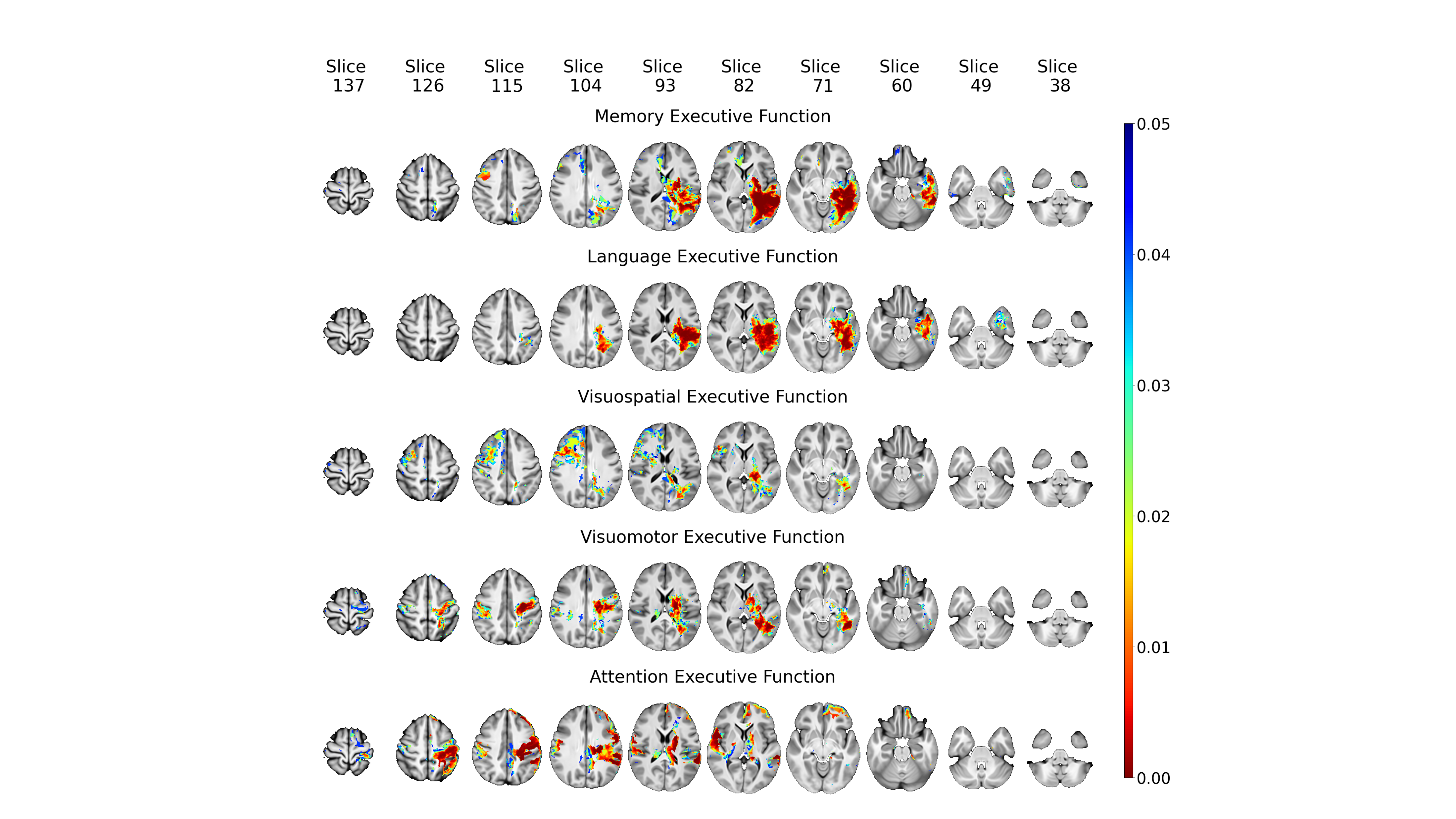

Supplement: vdae020_suppl_Supplementary_Data [file vdae020_suppl_supplementary_data.zip › Supplementary Material S6.tif]

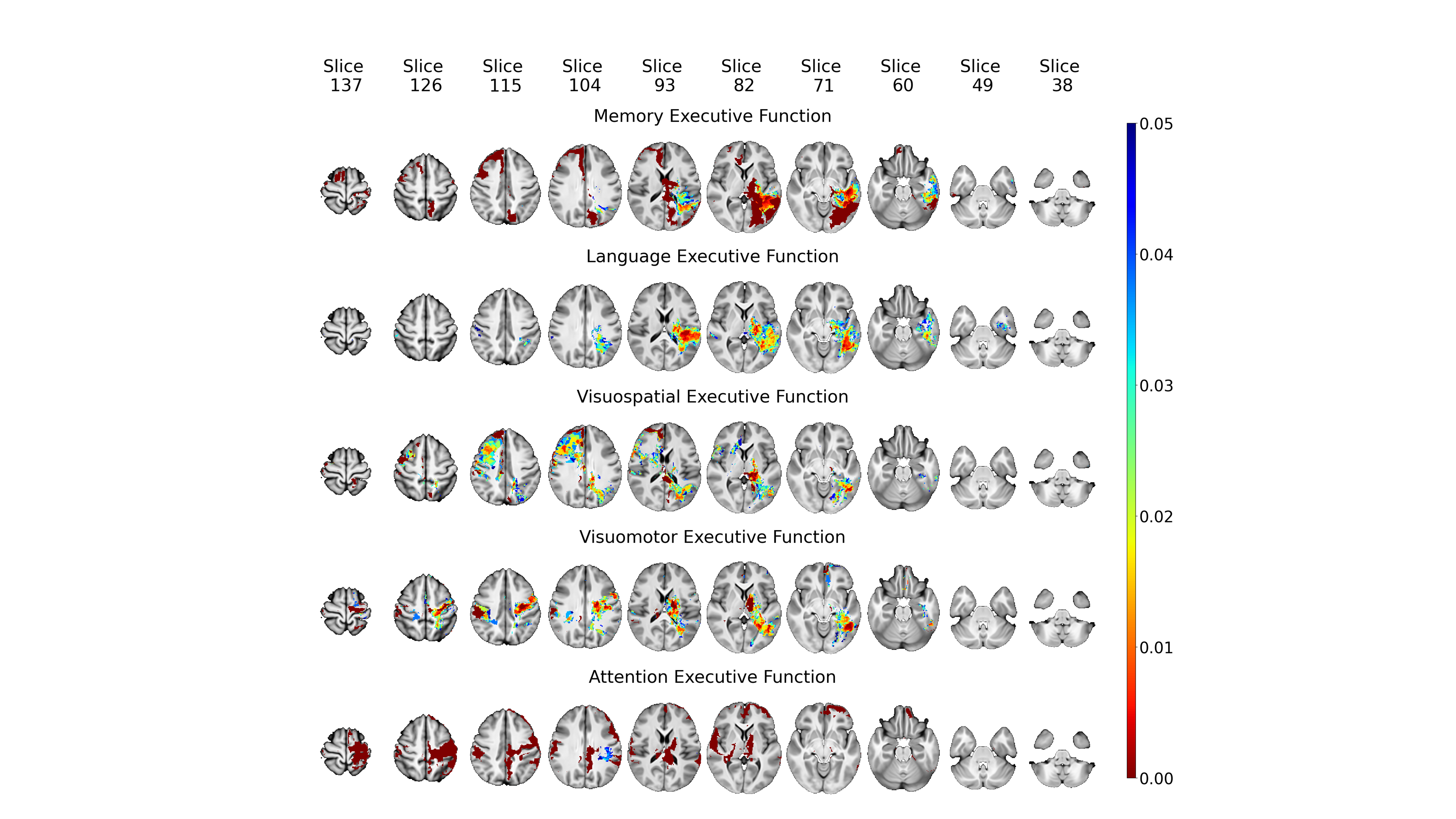

Supplement: vdae020_suppl_Supplementary_Data [file vdae020_suppl_supplementary_data.zip › Supplementary Material S7.tif]

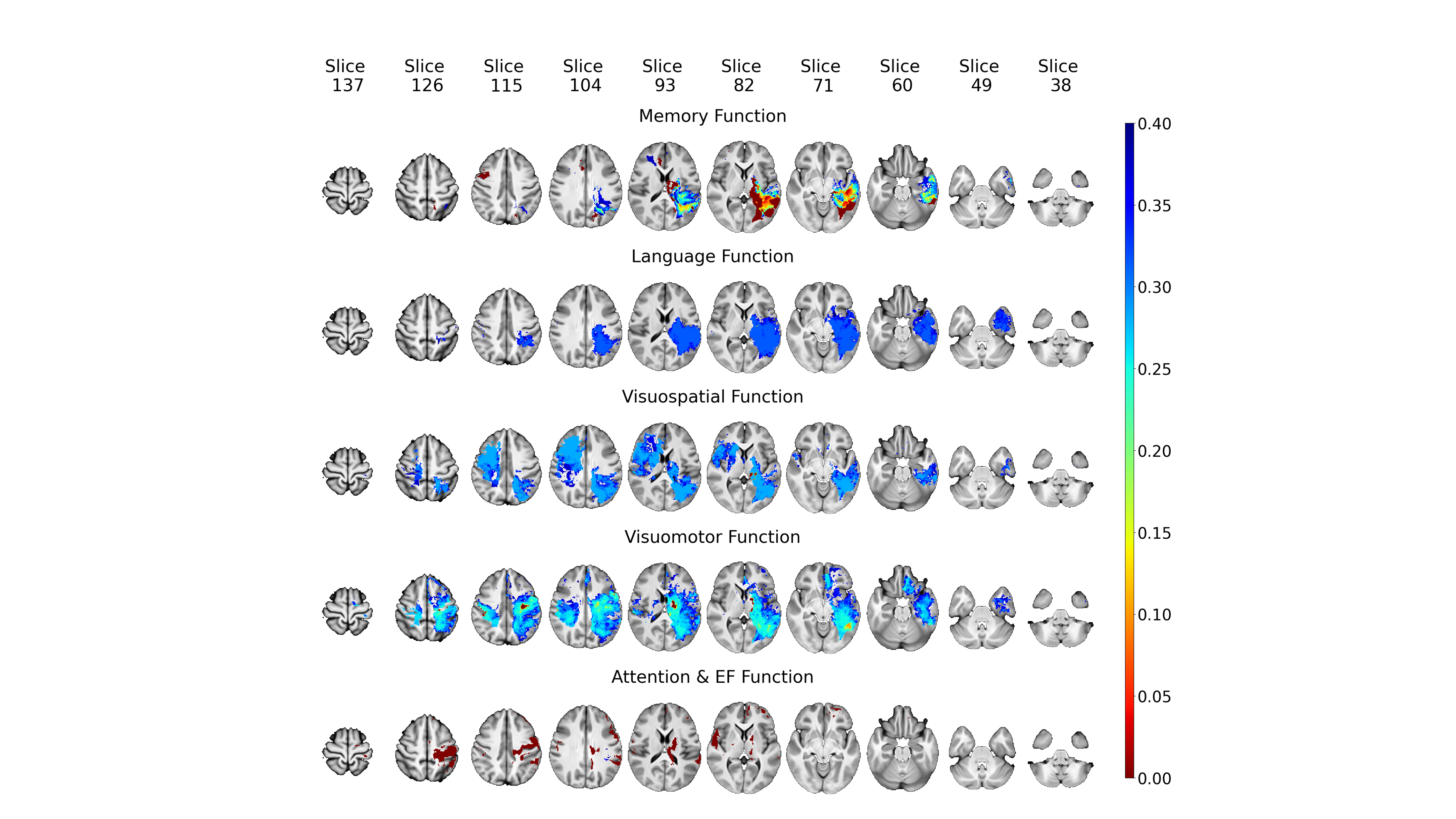

Supplement: vdae020_suppl_Supplementary_Data [file vdae020_suppl_supplementary_data.zip › Supplementary Material S8.tif]

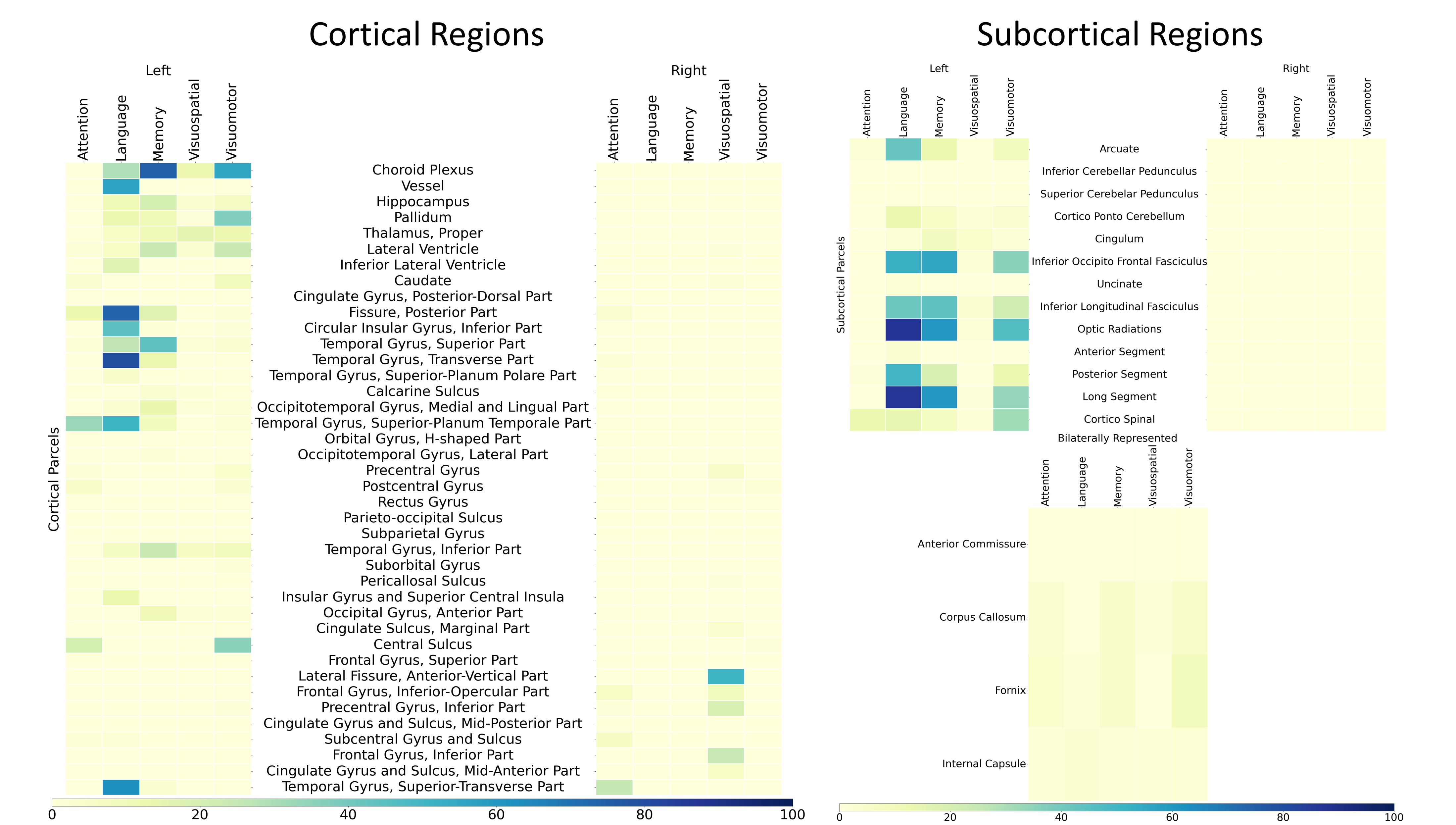

Supplement: vdae020_suppl_Supplementary_Data [file vdae020_suppl_supplementary_data.zip › Supplementary Material S9.tif]
